# Development of genome-wide SSR markers through *in silico* mining of guava (*Psidium guajava* L.) genome for genetic diversity analysis and transferability studies across species and genera

**DOI:** 10.3389/fpls.2025.1527866

**Published:** 2025-04-25

**Authors:** Kritidipta Pramanik, Amit Kumar Goswami, Chavlesh Kumar, Rakesh Singh, Ratna Prabha, Shailendra Kumar Jha, Madhubala Thakre, Suneha Goswami, Kaustav Aditya, Avantika Maurya, Sagnik Chanda, Prabhanshu Mishra, Shilpa Sarkar, Ankita Kashyap

**Affiliations:** ^1^ Division of Fruits and Horticultural Technology, ICAR-Indian Agricultural Research Institute, New Delhi, India; ^2^ Division of Genomic Resources, ICAR- National Bureau of Plant Genetic Resources, New Delhi, India; ^3^ Agricultural Knowledge Management Unit, ICAR-Indian Agricultural Research Institute, New Delhi, India; ^4^ Division of Genetics, ICAR-Indian Agricultural Research Institute, New Delhi, India; ^5^ Division of Biochemistry, ICAR- Indian Agricultural Research Institute, New Delhi, India; ^6^ Division of Agricultural Statistics, ICAR- Indian Agricultural Statistics Research Institute, New Delhi, India; ^7^ Division of Molecular Biology and Biotechnology, ICAR- Indian Agricultural Research Institute, New Delhi, India; ^8^ Department of Horticulture, PGCA, Dr. Rajendra Prasad Central Agricultural University, Pusa, Bihar, India

**Keywords:** guava, *in silico*, FHTGSSRs, polymorphism information content, cross-species transferability

## Abstract

Guava (*Psidium guajava* L.) is one of the economically major fruit crops, abundant in nutrients and found growing in tropical-subtropical regions around the world. Ensuring sufficient genomic resources is crucial for crop species to enhance breeding efficiency and facilitate molecular breeding. However, genomic resources, especially microsatellite or simple sequence repeat (SSR) markers, are limited in guava. Therefore, novel genome-wide SSR markers were developed by utilizing chromosome assembly (GCA_016432845.1) of the “New Age” cultivar through GMATA, a comprehensive software. The software evaluated about 397.8 million base pairs (Mbp) of the guava genome sequence, where 87,372 SSR loci were utilized to design primers, ultimately creating 75,084 new SSR markers. After *in silico* analysis, a total of 75 g-SSR markers were chosen to screen 35 guava genotypes, encompassing wild *Psidium* species and five jamun genotypes. Of the 72 amplified novel g-SSR markers (FHTGSSRs), 53 showed polymorphism, suggesting significant genetic variation among the guava genotypes, including wild species. The 53 polymorphic g-SSR markers had an average of 3.04 alleles per locus for 35 selected guava genotypes. Besides, in this study, the mean values recorded for major allele frequency, gene diversity, observed heterozygosity, and polymorphism information content were 0.73, 0.38, 0.13, and 0.33, respectively. Among the wild *Psidium* species studied, the transferability of these novel g-SSR loci across different species was found to be 45.83% to 90.28%. Furthermore, 17 novel g-SSR markers were successfully amplified in all the selected *Syzygium* genotypes, of which only four markers could differentiate between two *Syzygium* species. A neighbour-joining (N-J) tree was constructed using 53 polymorphic g-SSR markers and classified 35 guava genotypes into four clades and one outlier, emphasizing the genetic uniqueness of wild *Psidium* species compared to cultivated genotypes. Model-based structure analysis divided the guava genotypes into two distinct genetic groups, a classification that was strongly supported by Principal Coordinate Analysis (PCoA). In addition, the AMOVA and PCoA analyses also indicated substantial genetic diversity among the selected guava genotypes, including wild *Psidium* species. Hence, the developed novel genome-wide genomic SSRs could enhance the availability of genomic resources and assist in the molecular breeding of guava.

## Introduction

Guava (*Psidium guajava* L.), a member of the *Psidium* genus, is an economically important fruit crop commercially cultivated in pan-tropical regions. It possesses a diploid chromosome number of 2n = 22 and a genome size of around 450 Mbp (Coser et al., 2012; Feng et al., 2021). Guava fruit contains substantial amounts of Vitamin A, Vitamin C, and Vitamin B complex, and mineral nutrients like iron, calcium, zinc, potassium, dietary fibres, and pectin (Hassimotto et al., 2005; Vijaya et al., 2020; Jamieson et al., 2022). Additionally, it also contains phenolic fractions viz., caffeic, catechin, ellagic, p-coumaric, rutin, and trans-cinnamic acids in different developmental stages and serves as bioactive compounds that have anti-diabetic, antioxidant, anti-cancer, and anti-inflammatory properties (Flores et al., 2015; Dos Santos et al., 2017; Shukla et al., 2021). Moreover, its seeds contain around 5-13% oil, which consists of a substantial amount of omega-3 and omega-6 fatty acids (Adsule and Kadam, 1995). Though it was brought to India by the Portuguese in the 17^th^ century, guava has naturalized under Indian conditions due to its wider edaphic and climatic adaptability, and today India is a major guava-producing country globally, with an annual production of 5.35 million metric tons on approximately 358 thousand hectares of land (Ministry of Agriculture & Farmers Welfare (MoA&FW), 2024-25).

Due to the rising demand for guava fruit among health-conscious people, varietal developmental programs in guava have set different features viz., good fruit size with uniform shape, thick pulp with a small seed core that is embedded with fewer seeds having soft seed coats, good storage life, attractive peel and pulp colours, dwarf stature, high yielding efficiency, and tolerance/resistance to wilt and nematodes (Ray, 2002; Dinesh and Iyer, 2005; Dinesh and Vasugi, 2010; Kumar et al., 2020). Moreover, it is one of the perennial fruit trees and due to its allogamous nature (41% cross-pollination), its genetic background remains mostly heterozygous (Morton, 1987). Therefore, the trait-specific genetic improvement of guava is tedious and time-consuming through classical breeding (Thakur et al., 2021). These challenges in traditional breeding can be surmounted by utilizing biotechnological approaches, especially genomics such as genomic-assisted breeding (GAB) and marker-assisted breeding (MAB) which expedites guava improvement programs through the selection of genotypes at the seedling stage for traits of interest (Varshney et al., 2005; Kole et al., 2015; Baumgartner et al., 2016; Thakur et al., 2021). Molecular markers are key genomic tools in genetics and breeding that have been explored and utilized at every step of varietal development programs, including the evaluation of germplasm (Valdes-Infante et al., 2003; Rodriguez et al., 2007), trait-specific association mapping studies (Sheetal, 2010), hybridity estimation (Rao et al., 2008), QTL identification, linkage map construction, and marker-assisted breeding (Ritter, 2012; Maan et al., 2023). Among the two types of molecular markers (dominant and co-dominant), co-dominant markers can differentiate between homozygous and heterozygous individuals (Zhao and Kochert, 1993). Therefore, they are preferred in genetics and breeding studies of many crop species. Among the co-dominant DNA-based markers, SSR or microsatellite markers are the most accepted robust markers in crop species due to their easy scoring, high reproducibility, and high cross-species transferability (Collard et al., 2008; Kumar et al., 2020). While EST-SSR markers are valuable for genetic analysis, they come with certain limitations like low polymorphism levels and a tendency to concentrate in gene-rich regions of the genome, which may restrict their utility, especially in constructing linkage maps (Temnykh et al., 2000). Here, the importance of genome-wide SSR markers (g-SSR) increases in such analyses due to their whole genome coverage (Powell et al., 1996). Despite these advantages, only a limited number of g-SSR markers are reported in guava, and barely a small set of validated g-SSR markers are present in the public domain (Kumar et al., 2020; Thakur et al., 2021; Kumar et al., 2023).

Previously, microsatellite markers were developed by constructing microsatellite-enriched libraries involving selective hybridization including standard procedures for identifying microsatellite sequences using biotin-labelled probes (Edwards et al., 1996; Kumar et al., 2020). This method is robust and reproducible, but time-consuming and costly (Santana et al., 2009; Luo et al., 2020). Nowadays, next-generation sequencing (NGS) helps uncover the complete genome structure of various crops, improving our understanding of developing molecular markers. NGS has been used for sequencing many fruit trees like *Vitis vinifera* (Jaillon et al., 2007), *Carica papaya* (Ming et al., 2008), *Malus* × *domestica* (Velasco et al., 2010), *Fragaria vesca* (Shulaev et al., 2011; Hirakawa et al., 2014), *Prunus mume* (Zhang et al., 2012), *Prunus persica* (Verde et al., 2013), *Pyrus bretschneideri* (Wu et al., 2013; Chagné et al., 2014), and *Mangifera indica* (Wang et al., 2020). The genome sequence information is also available for Myrtaceae family members, including *Eucalyptus grandis* (Myburg et al., 2014), *Leptospermum scoparium* (Thrimawithana et al., 2019), Chinese guava cultivar “New Age” (Feng et al., 2021) and Indian guava cultivar “Allahabad Safeda” (Thakur et al., 2021) in the NCBI database. The genomic resources in guava are available in the form of chromosome assembly, draft sequences, RNA sequences, etc., in the NCBI database, which could serve as a basis for the identification and development of microsatellite markers. Moreover, various bioinformatical softwares have been developed for automated SSRs detection and the development of microsatellite markers using these sequences viz., TRF (Benson, 1999), MISA (Beier et al., 2017), and SciRoko (Kofler et al., 2007). But these tools often have long runtimes or cannot handle whole-genome analyses (Sharma, 2007). Furthermore, the statistical analyses provided by software like TROLL (Castelo et al., 2002) are very limited. Some tools, such as SSRLocator (Maia et al., 2008), are platform-specific and only run on Microsoft Windows, which is not ideal for handling large datasets. Command-line tools like MISA (Beier et al., 2017) lack graphical interfaces, posing difficulties for non-bioinformaticians. Moreover, CandiSSR (Xia et al., 2016) and SSRPoly (Duran et al., 2013) are inefficient for marker design due to their slow performance and reliance on existing primer design tools. Despite the availability of many tools, none provide a complete set of operations for identifying “SSRs”, analysing these “SSRs” ‘ distribution across the entire genome, designing SSR primer pairs, and polymorphism screening of developed markers through e-PCR algorithm like GMATA (Wang and Wang, 2016). Therefore, mining these genomic sequences, identifying the microsatellite sites, developing genome-wide microsatellite markers, and validation through diversity studies including their cross-species and genus transferability is targeted in the present study.

## Materials and methods

### SSR mining and statistical analysis of identified SSRs

For genome-wide SSR mining, the chromosome assembly of *Psidium guajava* cultivar “New Age” (Feng et al., 2021) - GCA_016432845.1 was used as a reference database, and it was downloaded from the NCBI database in FASTA format (https://www.ncbi.nlm.nih.gov/datasets/genome/GCA_016432845.1/). This sequence was used as an input for GMATA (Genome-wide Microsatellite Analysing Toward Application) software (Wang and Wang, 2016). To enhance SSR mining, GMATA implemented a technique that entailed partitioning the sequence (which had a default length exceeding 2 Mbp) into smaller segments to expedite the operation. To ensure accurate identification of SSRs at the sequence borders, a brief overlapping region of 20 base pairs (bp) was added to the end of each segment by default. The SSR units, consisting of nucleotides G, C, A, and T, were calculated dynamically as a motif library of specified lengths using metacharacters and a regular expression patterning method in Perl version 5. Perl’s pattern-matching method was utilized to identify recurring patterns, and the obtained data was then employed to generate information regarding SSR loci. After identifying SSRs in the genomic DNA sequence, a separate output file was generated. It provided detailed information on the SSR loci, including their start and finish positions, the number of repetitions, and the motif on each chromosome. Using this file, the statistical module of this software generated a new file that provides statistical information about motif type, motif composition, grouped complementary motifs, SSR distribution, and SSR length. This study specifically focused on motif type, motif composition, and SSR length for *in silico* analysis.

### Designing of markers and *in silico* polymorphism scoring

The primer designing module of GMATA used the SSR loci along with their original DNA sequences to generate primer pairs. The marker was mapped digitally with simulated PCR using e-PCR in GMATA to generate the amplicon and identify allele sizes. This e-mapping module required the presence of a DNA sequence file of *Psidium guajava* and a marker file as inputs. Upon executing the application, a report file was generated, which includes detailed information regarding the e-PCR findings of markers for each chromosome. Polymorphism was evaluated by assigning scores to amplified fragments from each marker in the genome according to their size. Furthermore, a summary report was also generated, offering a detailed analysis of allele distribution for all markers, the total sequences with mapped markers, the overall number of mapped markers, the total number of amplified fragments, and the average number of fragments per mapped marker.

### Synthesis of primer pairs

After *in silico* analysis, a total of 75 markers were selected from 11 chromosomes of guava for further analysis. At least five primer pairs (forward & reverse primers) from each chromosome were selected. Some important factors were considered during the selection of primer pairs *viz.*, the microsatellite regions should have at least five or more motif repeats, the GC content of the selected primers should be between 40 to 60%, and the annealing temperature (T_a_) of the primer pairs should range from 56 to 60°C. After synthesizing the novel g-SSR (FHTGSSRs) primer pairs, a stock solution of 100 pmol was also prepared based on their molecular weight by adding the required amount of TE buffer. To prepare a working sample, 10μl of each forward and reverse primer stock solution was mixed with 90μl of Milli-Q^®^ lab water separately in a 1.5 ml centrifuge tube. Finally, the diluted and stock samples were stored at 4°C and -20°C respectively.

### Plant materials

A total of 35 guava genotypes, including commercial guava cultivars, exotic varieties, and wild *Psidium* species, were selected for diversity and cross-species transferability studies. Besides this, three accessions of *Syzygium cumini* and two accessions of *Syzygium fruticosum* species were also chosen to confirm the cross-genera transferability of newly designed genome-wide SSRs (**Table 1**). The leaf samples from the selected guava genotypes were collected from the guava field gene bank at the Division of Fruits and Horticultural Technology, ICAR-IARI, New Delhi (28°38’54”N,77°09’12”E), whereas leaves of five jamun genotypes were collected from the plantation area of the IARI campus, New Delhi (28°38’25.0”N, 77°09’54.2”E).

**Table 1:** List of selected genotypes.

S. No.	Genotype	Type
1	Allahabad Safeda	Commercial Cultivar
2	Allahabad Safeda Variant	Cultivar
3	Hisar Safeda	Commercial Cultivar
4	L-49	Commercial Cultivar
5	Shweta	Commercial Cultivar
6	Thai	Commercial Cultivar
7	Pusa Pratiksha	Cultivar
8	Pant Prabhat	Cultivar
9	Red Selection	Cultivar
10	Lalit	Commercial Cultivar
11	Punjab Pink	Cultivar
12	Hisar Surkha	Commercial Cultivar
13	Red Diamond	Cultivar
14	Arka Kiran	Commercial Cultivar
15	Pusa Aarushi	Cultivar
16	Purple Guava	Cultivar
17	Guava Seedling-1	Unknown seedling
18	Guava Seedling-2	Unknown seedling
19	Guava Seedling-3	Unknown seedling
20	Guava Seedling-4	Unknown seedling
21	Guava Seedling-5	Unknown seedling
22	Seedling of Hong Kong White	Exotic cultivar
23	Seedling of Pink Acid	Exotic cultivar
24	Seedling of Thailand Seedless	Exotic cultivar
25	Seedling of Pear	Exotic cultivar
26	Seedling of 138-T	Exotic cultivar
27	Seedling of Gushiken Sweet	Exotic cultivar
28	Seedling of Klom Amporn	Exotic cultivar
29	*Psidium quadrangularis*	Wild species
30	*Psidium cattleianum*	Wild species
31	*Psidium cattleianum* variant	Wild species
32	*Psidium pumilum*	Wild species
33	*Psidium pumilum* variant	Wild species
34	*Psidium molle*	Wild species
35	*Psidium guineense*	Wild species
36	*Syzygium cumini* Seedling*-1*	Jamun Seedling
37	*Syzygium cumini* Seedling*-2*	Jamun Seedling
38	*Syzygium cumini* Seedling*-3*	Jamun Seedling
39	*Syzygium fruticosum* Seedling-1	Jamun Seedling
40	*Syzygium fruticosum* Seedling-2	Jamun Seedling

### Genomic DNA extraction and quality check

Three to four fresh, young, tender, and disease-free leaves were collected from each selected guava and jamun genotype. After that, the leaves were cleaned with 70% alcohol, wiped with tissue paper, wrapped in aluminium foil, and brought to the laboratory in cold boxes. The samples were properly labelled and stored at –80°C in a deep freezer until DNA extraction. DNA extraction of genotypes was done through the CTAB method with slight modification (Doyle and Doyle, 1987), where the DNA extraction buffer consisted of 2.5% w/v CTAB, 1M Tris HCl (pH 8.0), 0.5 M EDTA (pH 8.0), 4M NaCl, 3% PVP w/v, and 0.3% Beta-mercaptoethanol. A NanoDrop™ spectrophotometer (Thermo Fisher, USA) was used to measure the DNA concentration of genotypes. DNA samples with an A260/280 ratio between 1.7 to 1.9 and a concentration >250 ng/µL were selected for this investigation. Besides, the quality of the isolated genomic DNA was checked on 0.8% agarose gel. After assessing the quantity and quality of each DNA sample of the selected genotypes, the DNA concentration was diluted with TE buffer to achieve a working concentration of 20 ng/µl. The diluted samples were kept at 4°C for immediate use, while the stock of primers was stored at -20°C until further use.

### SSR profiling

A ready-to-use reaction mixture-OnePCR™ (GeneDireX, Inc), which contains Taq DNA polymerase, PCR buffer, dNTPs, and loading dye, was utilized to optimize the PCR conditions. The annealing temperature for each g-SSR primer pair was determined using a gradient PCR process where the annealing temperature was kept between 50 to 60°C for one minute. Then PCR conditions for SSR profiling were set on an initial denaturation at 94°C for five minutes, followed by 36 cycles of denaturation at 94°C for 30 seconds, annealing at a temperature specific to each SSR primer (determined by gradient PCR), extension at 72°C for one minute, and a final extension at 72°C for 10 minutes. The final reaction mixture for the PCR process was maintained at 16μl, comprising 2.5μl of genomic DNA, 6μl Master Mix (1x), 1μl each forward and reverse primers, and 5.5μl nuclease-free double-distilled water. The resulting PCR amplified products and a DNA ladder (DNAmark™ 100 bp) were loaded onto a 2.5% agarose gel containing ethidium bromide (18μl/500 ml) in 1x TAE buffer. The gel electrophoresis was run at a steady voltage of 5 V/cm for about three hours. The DNA profiles were then visualized under a gel documentation system (Gel Luminax, Zenith).

### Data scoring and analyses

The amplicon of each SSR was scored visually among the selected genotypes. All SSRs were assessed for clear, reproducible, and distinct monomorphic and polymorphic bands. Genetic diversity indices, including major allele frequency (MAF), allele frequency, allele count (A_n_), gene diversity (GD) or expected heterozygosity (H_e_), observed heterozygosity (H_o_), and polymorphism information content (PIC) for each g-SSR, were calculated with Power Marker v3.25 (Liu and Muse, 2005). A Neighbor-Joining (NJ) tree was also generated using Power Marker v3.25 (Liu and Muse, 2005). Additionally, the population structure analysis of guava genotypes was performed by Structure v2.3.4 (Pritchard et al., 2000). Population structure was determined through Structure Harvester (Earl and VonHoldt, 2012) using the Evano method. Moreover, GenAlEx v6.5 (Peakall and Smouse, 2006) was used for the Analysis of Molecular Variance (AMOVA) and Principal Coordinate Analysis (PCoA) of the guava genotypes.

## Results

### SSR mining and statistical analysis of SSRs

**Figure 1:**
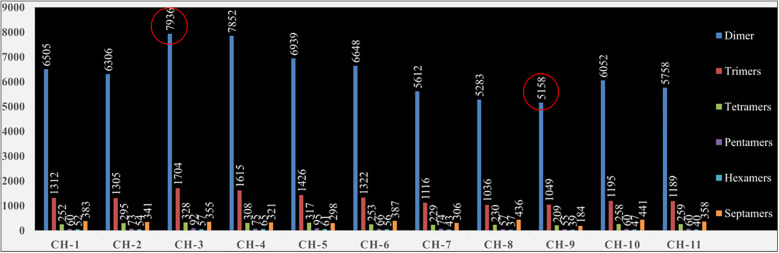
Chromosome wise distribution of dimers, trimers, tetramers, pentamers, hexamers and septamers repeats in guava genome.

A total of 397.8 Mbp of the guava genome sequence was analysed using the GMATA software, and 92,404 SSR loci were identified (**Supplementary Table 1**). The number of chunking sites varied from 11 (chromosome 9) to 17 (chromosomes 3 and 4), while the total number of SSR loci per chromosome varied from 6696 (chromosome 9) to 10475 (chromosome 3). The dimer-type motifs (>75%) were found to be the highest, followed by trimer-type motifs (>15%) in every chromosome of the guava genome (**Figure 1**). Among dimers, mostly “TC” (~13.5%) motif compositions were identified in every chromosome except chromosome-1 and chromosome-11, where the most common type motif was “GA” (~13.7%) and “AG” (~12.9%), respectively. Among trimers, mostly “AAT” (~1.4%) motif compositions were found in every chromosome (**Supplementary Figure 2**).

### Selection of primer pairs through *in silico* analysis

**Figure 2:**
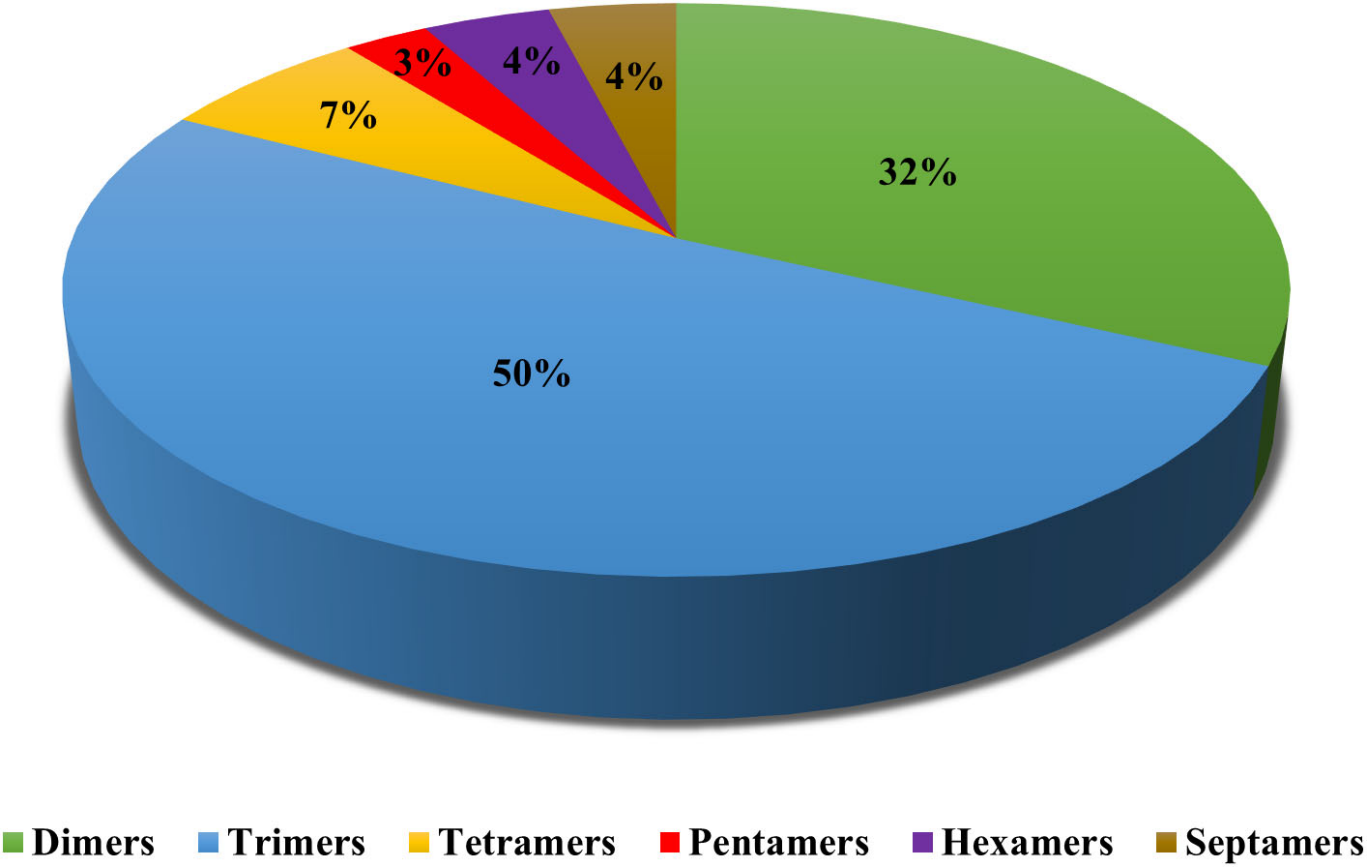
The percentage of various motifs considered during the selection of 75 g-SSR markers.

A total of 87,372 SSR loci were used by GMATA software for SSR markers designing, resulting in 75,084 novel SSR markers mined, spanning the genome of guava. Using the e-PCR algorithm, the average number of alleles was 2.78 per locus for the 75,084 SSRs. The highest and lowest numbers of markers were observed in chromosome 3 (8613) and chromosome 9 (5489), respectively (**Supplementary Table 2**). The highest average number of alleles per SSR marker was registered in chromosome 4 (3.20 alleles/SSR marker). After *in silico* analysis and genome-wide SSR mining, 75 novel g-SSR markers were selected for this study where trimer motif repeats were mostly used (**Figure 2**; **Supplementary Figure 3**).

### Screening of novel g-SSR markers

Of the 75-novel g-SSR markers, 72 showed reliable amplification in gradient PCR and were considered suitable for molecular profiling of guava genotypes due to their consistent amplification patterns. The amplification size of alleles varied between 110 to 620 bp amongst the selected g-SSR primer pairs (**Table 2**). However, three g-SSR markers showed no satisfactory amplification, even after repetitive PCR reaction optimisation attempts. All the selected wild species showed cross-species transferability for 28 g-SSR markers (FHTGSSR-3.1, FHTGSSR-3.2, FHTGSSR-2.3, FHTGSSR-3.4, FHTGSSR-3.5, FHTGSSR-3.8, FHTGSSR-4.4, FHTGSSR-4.5, FHTGSSR-4.6, FHTGSSR-5.1, FHTGSSR-5.4, FHTGSSR-5.5, FHTGSSR-5.6, FHTGSSR-5.8, FHTGSSR-6.2, FHTGSSR-6.7, FHTGSSR-6.9, FHTGSSR-7.1, FHTGSSR-7.4, FHTGSSR-7.5, FHTGSSR-7.9, FHTGSSR-8.2, FHTGSSR-8.4, FHTGSSR-9.1, FHTGSSR-9.2, FHTGSSR-10.3, FHTGSSR-11.3, FHTGSSR-11.5). In contrast, a total of 55, 43, 33, 64, 65, 62 and 63 novel g-SSR markers were amplified in *P*. *quadrangularis*, *P*. c*attleianum*, *P*. *cattleianum* variant, *P*. *pumilum*, *P*. *pumilum* variant, *P*. *molle* and *P*. *guineense* respectively (**Supplementary Table 3**).

**Table 2:** Information about 72 novel amplified g-SSR markers.

S. No	Marker Name	C.N	Marker Sequence	T_a_(°C)	Motif	Repetitions	Expected Product Size (bp)	Observed Product Size(bp)	Remark
1	FHTGSSR-1.2	1	F-GCAAAAGGTGGGATCTAGCA R-CAGTCTTCCAGGGACACCAT	59	AAG	6	277	290-300	Polymorphic
2	FHTGSSR-1.3	1	F-GGGAGAGGGAGAGAAAATGG R-CAAATGAGCCAAAACAGCAA	55	AG	25	364	290	Monomorphic
3	FHTGSSR-1.4	1	F- AAAACAGTCGGGTGCATTTC R-CAGGAGGCAGTCACCTTAGC	59	TC	5	367	360-380	Polymorphic
4	FHTGSSR-1.5	1	F-GTGAAGGAAGCTTGGATGGA R-TGATGCTTGAAAATGCGAAC	52.6	AATAT	7	395	380-440	Polymorphic
5	FHTGSSR-2.1	2	F-ATGAAACAGCGGATGAACCT R-TCACCCAGCGAAACCTTATC	59.2	GA	20	157	140-200	Polymorphic
6	FHTGSSR-2.2	2	F-CCGACACGCCATTAGAAGAT R-TAGCGTGCACAGAATTACGG	52.6	GAG	5	360	350-370	Polymorphic
7	FHTGSSR-2.3	2	F-TCGAAACCACAACCAACAAA R-ACCTGCATGTGGCTTTTACC	55	CAA	5	139	140	Monomorphic
8	FHTGSSR-2.4	2	F-ACGAATCGAGTCTTGGCATT R-CGCAATTGGATTGATGTTTG	50.7	TC	32	338	250-370	Polymorphic
9	FHTGSSR-2.5	2	F-TCAGGCATTCTGGTGATGAA R-TCGATGAACCCAAAGTGAAA	59.2	TTTA	6	288	290	Monomorphic
10	FHTGSSR-2.6	2	F-GACGAAGGCGAAGATGAAGA R-GCGACAAATCACACAAAAGG	65	GAAGAT	5	273	280-380	Polymorphic
11	FHTGSSR-3.1	3	F-GTCGAAGAGATCAGGGCATC R-AAACAGCCCAGCAATTCATC	50.7	AGC	5	378	350-380	Polymorphic
12	FHTGSSR-3.2	3	F-ACACCCGTGCAAGAAGAAGT R-GATGGGCTTTAGTGGGTTGA	59.2	CAG	5	309	300-400	Polymorphic
13	FHTGSSR-3.3	3	F-GGAAAGAGTGCGAATTACGG R-GCTGGAGAATTGGATTGGAA	59.2	CCT	8	342	360-370	Polymorphic
14	FHTGSSR-3.4	3	F-CTCCCATCCTCTGTCTCTGC R-CACGAGAAGGGGCTTTACTG	58.3	CTCCCT	5	330	340-350	Polymorphic
15	FHTGSSR-3.5	3	F-CGACTTTTGGGTGAAAGGAA R-ACTTTGCTTGGTGAGGGAAA	58.3	GA	10	340	360-380	Polymorphic
16	FHTGSSR-3.6	3	F-TTCCGACAGCGTCAAGAATA R-CATGCAATCAGGCAGAGAGA	59.2	AG	16	214	190-210	Polymorphic
17	FHTGSSR-3.7	3	F-CAAAGGGTAGGTGGGGAAAT R-ATCAGGAAGGACGCTGAAGA	59.2	TC	21	398	380-420	Polymorphic
18	FHTGSSR-3.8	3	F-GGGTTAGAGCGTCGTGACAT R-GCTGTTGATGCAAGTGGAGA	59.2	TAA	14	226	210-280	Polymorphic
19	FHTGSSR-3.9	3	F-AGCGGCAACATCAAGAAGAT R-CCTTCTATTCGGTTCCGTGA	45.7	AAT	13	367	360	Monomorphic
20	FHTGSSR-4.1	4	F-CCCATTCTCTTGCATCGAGT R-GCAATGTTCTTACGCCAACA	59.2	GCCAGTT	5	366	340-380	Polymorphic
21	FHTGSSR-4.2	4	F-CAAATCAGCGACTCAACCAA R-AAATGTCGTCGTCCTCTTCG	59.2	GCA	6	395	400-440	Polymorphic
22	FHTGSSR-4.3	4	F-GCTTAGGTGTGCTCCTGGTC R-GGTCTCGGATGCAATCAACT	58.3	TGTA	6	200	210	Monomorphic
23	FHTGSSR-4.4	4	F-TTCAATTTCGGGTTGACACA R-AACCCACTTTCTAGGCAGCA	58.3	CT	11	279	280-340	Polymorphic
24	FHTGSSR-4.5	4	F-TCATCGGACATTCCAAGACA R-TTGCACCAAAGACAGTTTGC	59.2	GA	28	259	200-280	Polymorphic
25	FHTGSSR-4.6	4	F-CGTGCCCTTTATGACCCTTA R-ATGCCACTAAGGTCCACGTC	59.2	TC	15	169	170-190	Polymorphic
26	FHTGSSR-4.7	4	F-CCTCTGACCTCGAGCTTGTC R-GACAACCTTGCTGGACCTGT	50	ATA	11	318	300-380	Polymorphic
27	FHTGSSR-4.8	4	F-CAACCGGCCAGAGGTATAGA R-GCCAAAGGAGGAAGAAGAGG	59.2	TTTC	9	363	350-360	Polymorphic
28	FHTGSSR-5.1	5	F-CCTGGCTCAAAGTTGGGATA R-ACAAGGCTGTGGGATGTTTC	51.5	TC	28	339	360-400	Polymorphic
29	FHTGSSR-5.2	5	F-CCATCTCCATCTCCATCTCC R-TTGTTCCCTCCTCAATCTCG	64.7	AAG	6	386	400	Monomorphic
30	FHTGSSR-5.4	5	F-CAGCGCATTCGACAGAAATA R-TGCTTGGAAGGCAATTATCC	51.5	AAT	5	397	400	Monomorphic
31	FHTGSSR-5.5	5	F-CGATCATCTACACCCGAACC R-CTACGTGAGGCCATTCAACA	50	ATTT	5	298	300	Monomorphic
32	FHTGSSR-5.6	5	F-TTTTACTGTTTGGGCCTTGG R-GCGCTCCTAATTGCATCTCT	45.7	AG	15	319	300-320	Polymorphic
33	FHTGSSR-5.7	5	F-GTTGCCACTACCCCAAGAGA R-ATCCAGGTTGTGAAGGATGC	50	GA	15	330	300-390	Polymorphic
34	FHTGSSR-5.8	5	F-TTCTCATTCAGGTGGGGTTC R-CTCCTCTTTCGATCGTCCAG	59.7	GAA	11	354	360	Monomorphic
35	FHTGSSR-5.9	5	F-GCGCAACAAGAATGGATGTA R-CCTCCTTGTGCTTTCTCCAC	59.7	TTA	12	360	320-380	Polymorphic
36	FHTGSSR-6.1	6	F-CGGATGCAACCTTTCATTTT R-CGGTTCAAAATCGGCACTAT	55	AAAT	5	154	120-240	Polymorphic
37	FHTGSSR-6.2	6	F-CTCCATCCCCACTCCAAGTA R-GCATTGGCGAAGTCCACTAT	50	AAT	6	388	390 = 410	Polymorphic
38	FHTGSSR-6.3	6	F-AAGCGGAGAAACCCTACGAT R-TGTTGGTGACGTTCTTTCCA	52.6	CT	16	358	320-380	Polymorphic
39	FHTGSSR-6.4	6	F-TACATGCCGTCAAGGTTTCA R-GCTTGCACGTTGGTTTCTCT	52.6	TC	12	257	340-360	Polymorphic
40	FHTGSSR-6.5	6	F-AGCCCATCGTCTCCTACCTT R-ACGGTCAGAACCCACAAGTC	57.2	AGG	7	354	380	Monomorphic
41	FHTGSSR-6.6	6	F-TAGCGAAGCAAGAGCATTGA R-ATGGCACGGTTTGGCTTACT	54	AT	11	247	210-280	Polymorphic
42	FHTGSSR-6.7	6	F-TGGGCTGAAGAAATCCACTC R-TTTTATTGTGGGCCCTTGAC	54	AG	10	331	340-360	Polymorphic
43	FHTGSSR-6.8	6	F-CTAGGTCAATTGCGGGGATA R-ACAGAAGACGAATGCCTGGA	57.2	TTA	11	319	300-360	Polymorphic
44	FHTGSSR-6.9	6	F-GGAAAGGCATTTTCGTCCTT R-CAACCTCTCGGAAGATTTGC	57.2	CTCCC	7	198	180-600	Polymorphic
45	FHTGSSR-7.1	7	F-TGCGACGGTATCGATGTAAG R-CCTGCCCGAATATAAAGCAA	52.6	TTC	7	176	180-190	Polymorphic
46	FHTGSSR-7.2	7	F-ACGATTTTAGATGCGCTCGT R-GGCTAGTTCATTTCGGCATC	57.2	AAT	6	266	280	Monomorphic
47	FHTGSSR-7.3	7	F-GGTGATCTACGTTCCGCATT R-CCTTTGCCATCCATGTCTTT	52.6	GT	7	384	380-400	Polymorphic
48	FHTGSSR-7.4	7	F-TTCGACACTCTTCGACATGC R-TGTGACACGACACGACACAT	52.6	AAG	6	379	180-400	Polymorphic
49	FHTGSSR-7.5	7	F-TGAAATCCCCATGACCTGTT R-ATGTGCAGGCATTTTGAGTG	50.7	CATCAA	5	394	110-390	Polymorphic
50	FHTGSSR-7.6	7	F-GACTAAGGACGTCGCGTTTC R-CCCGTTCAATCGACATTTCT	54	TA	17	238	220-270	Polymorphic
51	FHTGSSR-7.7	7	F-GGCTCAAGGAAGCACTGAAC R-GCGCGTCGATCTCTTTCTAC	54	GA	15	208	180-200	Polymorphic
52	FHTGSSR-7.8	7	F-GAAAGCGAACCTGGACTCAC R-TTCGTGGAATTCACCATTGA	57.2	TTA	15	308	280-320	Polymorphic
53	FHTGSSR-7.9	7	F-CTCCGATCGAAACCCTATCA R-CACACCTACGTCTGCTTGGA	57.2	CTT	7	313	310	Monomorphic
54	FHTGSSR-8.1	8	F-GCCAATTCCACTCGAAAATC R-CTCAACCACCTTTTGCTGCT	50.7	TC	7	351	360-380	Polymorphic
55	FHTGSSR-8.2	8	F-GGTTGCTTCCTGCATGATTT R-GAAAACCCCAACAGGACTGA	55.7	AAG	5	194	190-200	Polymorphic
56	FHTGSSR-8.3	8	F-CAAACCGACCCAATTTGAAG R-GGGTCAAACCAATGAATGTG	55.7	AAT	17	334	330	Monomorphic
57	FHTGSSR-8.4	8	F-CAGGATGCATGGTTTGACAG R-TCCAGACCAAACAGCAGAGA	52.6	TTA	5	388	400-600	Polymorphic
58	FHTGSSR-8.5	8	F-GGTAAGTTGCCGAAGGTTGA R-TGCGCAGCTTGATTTATTTG	52.6	CGG	8	357	360-380	Polymorphic
59	FHTGSSR-9.1	9	F-GCTGGGCGCTATTACTTGAG R-GTGACACGTGGCTTGTTGAC	59.2	TTA	14	128	100-140	Polymorphic
60	FHTGSSR-9.2	9	F-ACCAGTCGTGTTCCCTAACG R-CTAGGACGACCCCTGCATAA	55.7	TCT	5	376	380-400	Polymorphic
61	FHTGSSR-9.3	9	F-TCTGGCCAAAGAAAATCTGC R-GCCCATTATCACGCCTTAGA	54	AAT	5	364	370	Monomorphic
62	FHTGSSR-9.4	9	F-TGTCGGTGAAGAGCTTCCTT R-TTACTGTGCGACGTCCTCCT	55.7	GAA	5	214	220	Monomorphic
63	FHTGSSR-9.5	9	F-CGGACCTCGTGTCACCTTAT R-GGGAGGTAAATGAGTGGGCTA	59.2	TC	5	303	305	Monomorphic
64	FHTGSSR-10.2	10	F-ATGGGCTTGACTTTGACTGG R-GAGGGTGCGTTTATTCGAGA	59.7	TAA	5	341	350	Monomorphic
65	FHTGSSR-10.3	10	F-GAAAGGGGTCCAAGTTCCTC R-CCGCAGGCTTCTACTGTTTC	59.7	AAG	5	366	360-380	Polymorphic
66	FHTGSSR-10.4	10	F-GAGATTGGAACGCACCTGAT R-TACATAATGCCCATGGATGC	59.7	AAT	8	380	380-450	Polymorphic
67	FHTGSSR-10.5	10	F-GGTGCTTCTTCTTCGACCTG R-AAGCTCGTCCTCTCGACTTG	59.7	GAA	5	134	130	Monomorphic
68	FHTGSSR-11.1	11	F-CCCTAAACCCTAAACCCTAAACC R-GGAGAACATGTGTTTGGCTTATC	57.2	ACCCTAA	9	372	200-320	Polymorphic
69	FHTGSSR-11.2	11	F-CTTGGCAGTGTTACGTGTCG R-GGATGATAGCGCAGCCATAG	54	AG	6	303	280-350	Polymorphic
70	FHTGSSR-11.3	11	F-CTATGCCGGAGGTCATGTCT R-TTTTGGTGGAAACTCCATGTC	55.7	GA	8	400	400	Monomorphic
71	FHTGSSR-11.4	11	F-TAGGAGCGGTAGGTTTCACG R-TCGTTGACGTGCTAATCGTC	57.2	AAG	6	355	370-380	Polymorphic
72	FHTGSSR-11.5	11	F-TCCGGTGTTACAGGTCCTTC R-CATGCCGCTCACTTCAAATA	52.6	TTA	6	140	150-180	Polymorphic

Here, C.N., Chromosome Number; T_a_, Annealing Temperature

### Diversity analysis

**Figure 3:**
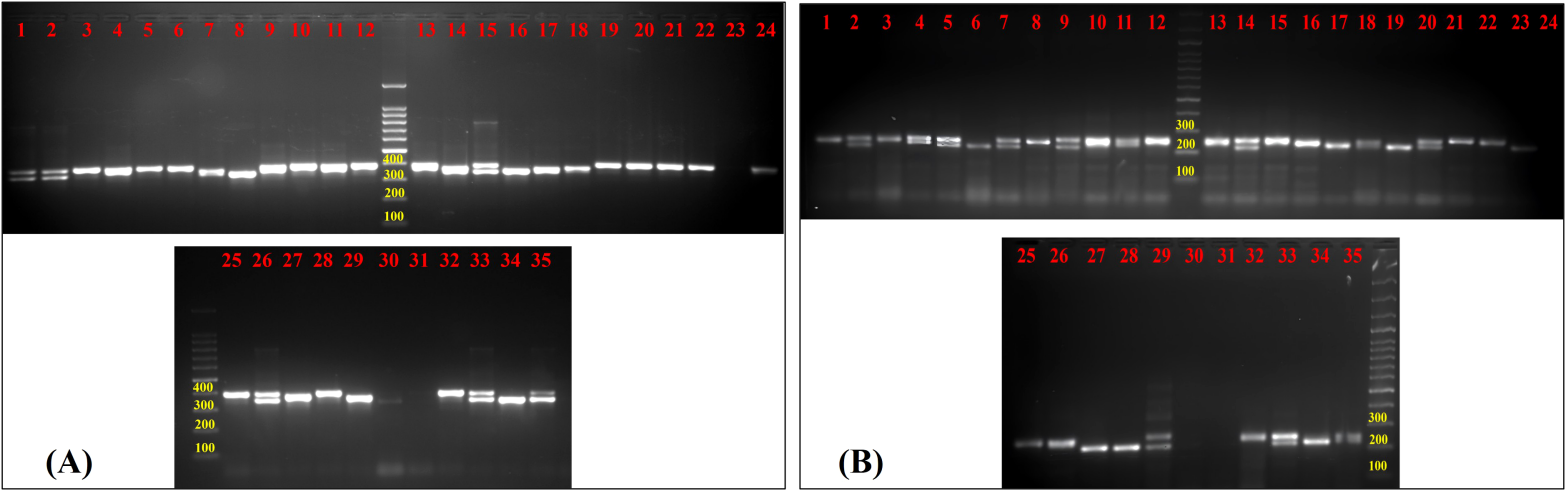
Gel image of amplified products of guava genotypes for **(A)** FHTGSRR-6.3 and **(B)** FHTGSRR-7.6. Here, (1) Allahabad Safeda, (2) Allahabad Safeda Variant, (3) Hisar Safeda, (4) L-49, (5) Shweta, (6) Thai, (7) Pusa Pratiksha, (8) Pant Prabhat, (9) Red Selection, (10) Lalit, (11) Punjab Pink, (12) Hisar Surkha, (13) Red Diamond, (14) Arka Kiran, (15) Pusa Aarushi, (16) Purple Guava, (17) Guava Seedling-1, (18) Guava Seedling-2, (19) Guava Seedling-3, (20) Guava Seedling-4, (21) Guava Seedling-5, (22) Hong Kong White Seedling, (23) Pink Acid Seedling, (24) Thailand Seedless Seedling, (25) Pear Seedling, (26)138-T Seedling, (27) Gushiken Sweet Seedling, (28) Klom Amporn Seedling, (29) *Psidium quadrangularis*, (30) *Psidium cattleianum*, (31) *Psidium cattleianum* variant, (32) *Psidium pumilum*, (33) *Psidium pumilum* variant, (34) *Psidium molle*, (35) *Psidium guineense*.

Out of 72 amplified novel g-SSRs, 53 markers were able to produce polymorphic amplicons (**Figure 3**; **Supplementary Figure 4**), while 19 markers were found monomorphic among the selected 35 guava genotypes. The guava genotypes were thoroughly molecularly characterised, and genetic diversity was analyzed using these 53 polymorphic g-SSRs. A total of 161 alleles were amplified by 53 g-SSR markers among the selected 35 guava genotypes. Each primer pair yielded 3.04 alleles on average, with the allele number ranging from 2 to 7 per locus. In this study, the highest PIC value was found 0.774 for FHTGSSR-4.5, though the average PIC for 53 polymorphic g-SSR markers was 0.331. Furthermore, average major allele frequency expected heterozygosity, and observed heterozygosity were measured at 0.725, 0.375, and 0.128 respectively (**Table 3**).

**Table 3:** Information about different diversity parameters of the 53 Polymorphic FHTGSSR loci.

Marker	MAF	Allele No.	H_e_	H_o_	PIC
FHTGSSR-1.2	0.970	2.0	0.059	0.000	0.057
FHTGSSR-1.4	0.561	2.0	0.493	0.879	0.371
FHTGSSR-1.5	0.813	3.0	0.320	0.125	0.294
FHTGSSR-2.1	0.688	4.0	0.480	0.281	0.435
FHTGSSR-2.2	0.853	2.0	0.251	0.000	0.219
FHTGSSR-2.4	0.618	5.0	0.571	0.176	0.534
FHTGSSR-2.6	0.576	3.0	0.549	0.606	0.468
FHTGSSR-3.1	0.857	3.0	0.251	0.057	0.231
FHTGSSR-3.2	0.871	3.0	0.230	0.086	0.213
FHTGSSR-3.3	0.938	2.0	0.117	0.063	0.110
FHTGSSR-3.4	0.657	2.0	0.451	0.000	0.349
FHTGSSR-3.5	0.886	2.0	0.202	0.114	0.182
FHTGSSR-3.6	0.452	3.0	0.620	0.387	0.541
FHTGSSR-3.7	0.688	3.0	0.477	0.125	0.427
FHTGSSR-3.8	0.457	4.0	0.639	0.114	0.567
FHTGSSR-4.1	0.758	3.0	0.389	0.000	0.347
FHTGSSR-4.2	0.938	2.0	0.117	0.000	0.110
FHTGSSR-4.4	0.543	4.0	0.616	0.000	0.559
FHTGSSR-4.5	0.271	6.0	0.802	0.114	0.774
FHTGSSR-4.6	0.657	2.0	0.451	0.000	0.349
FHTGSSR-4.7	0.344	7.0	0.758	0.125	0.720
FHTGSSR-4.8	0.750	2.0	0.375	0.000	0.305
FHTGSSR-5.1	0.743	3.0	0.389	0.086	0.324
FHTGSSR-5.6	0.929	2.0	0.133	0.086	0.124
FHTGSSR-5.7	0.471	5.0	0.655	0.147	0.595
FHTGSSR-5.9	0.656	4.0	0.502	0.063	0.443
FHTGSSR-6.1	0.970	3.0	0.059	0.061	0.058
FHTGSSR-6.2	0.529	2.0	0.498	0.029	0.374
FHTGSSR-6.3	0.636	4.0	0.545	0.182	0.503
FHTGSSR-6.4	0.859	2.0	0.242	0.094	0.212
FHTGSSR-6.6	0.897	4.0	0.191	0.059	0.183
FHTGSSR-6.7	0.757	3.0	0.397	0.171	0.363
FHTGSSR-6.8	0.515	4.0	0.614	0.273	0.546
FHTGSSR-6.9	0.515	6.0	0.658	0.500	0.614
FHTGSSR-7.1	0.941	2.0	0.111	0.000	0.105
FHTGSSR-7.3	0.853	2.0	0.251	0.000	0.219
FHTGSSR-7.4	0.941	3.0	0.112	0.059	0.109
FHTGSSR-7.5	0.371	4.0	0.723	0.200	0.673
FHTGSSR-7.6	0.455	3.0	0.622	0.364	0.543
FHTGSSR-7.7	0.818	3.0	0.312	0.000	0.288
FHTGSSR-7.8	0.652	2.0	0.454	0.697	0.351
FHTGSSR-8.1	0.515	2.0	0.500	0.000	0.375
FHTGSSR-8.2	0.765	2.0	0.360	0.000	0.295
FHTGSSR-8.4	0.957	3.0	0.083	0.029	0.081
FHTGSSR-8.5	0.906	2.0	0.170	0.000	0.155
FHTGSSR-9.1	0.691	3.0	0.469	0.235	0.418
FHTGSSR-9.2	0.912	2.0	0.161	0.000	0.148
FHTGSSR-10.3	0.943	2.0	0.108	0.000	0.102
FHTGSSR-10.4	0.971	3.0	0.058	0.059	0.057
FHTGSSR-11.1	0.806	3.0	0.329	0.129	0.302
FHTGSSR-11.2	0.530	5.0	0.562	0.030	0.472
FHTGSSR-11.4	0.875	2.0	0.219	0.000	0.195
FHTGSSR-11.5	0.914	2.0	0.157	0.000	0.144
Mean	0.725	3.038	0.375	0.128	0.331

Here, MAF, Major Allele Frequency; H_e_, Expected Heterozygosity; H_o_, Observed Heterozygosity; PIC, Polymorphism Information Content

### Phylogenetic relationship

**Figure 4:**
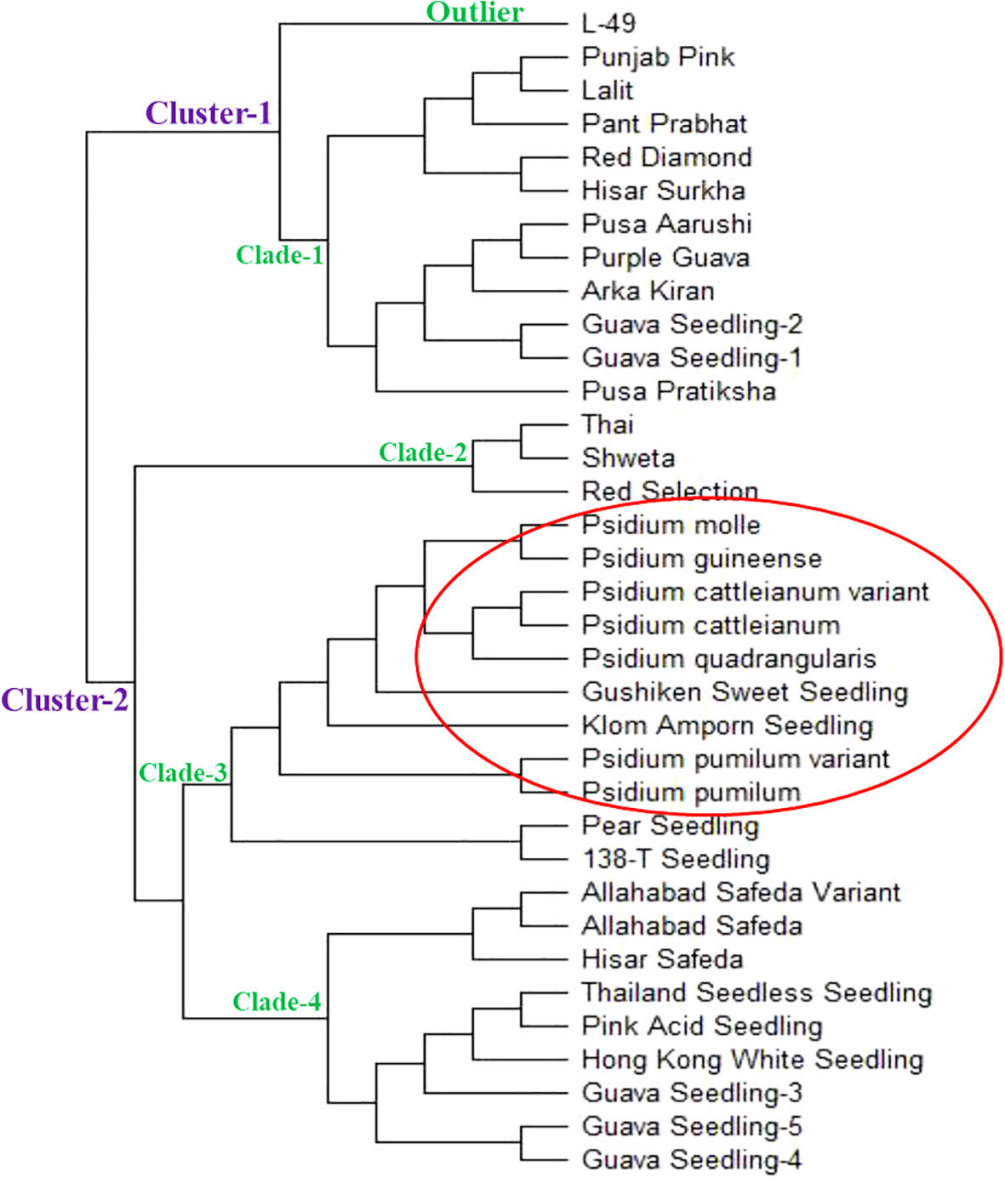
Neighbor- Joining tree of selected guava genotypes based on 53 polymorphic g-SSR markers.

The Neighbor joining (N-J) tree was constructed using the genotypic data from 53 polymorphic g-SSR markers, which grouped 35 selected guava genotypes into two major clusters (**Figure 4**). These were further simplified into four clades and one outlier. The clade-1 contained the guava genotypes viz., Punjab Pink, Lalit, Pant Prabhat, Red Diamond, Hisar Surkha, Pusa Aarushi, Purple Guava, Arka Kiran, Guava Seedling-1, Guava Seedling-2 and Pusa Pratiksha. The clade-2 comprised Thai, Shweta, and Red Selection genotypes. The clade-3 included the wild guava species and four exotic genotypes *viz.*, *Psidium pumilum*, *Psidium pumilum* variant, *Psidium cattleianum*, *Psidium cattleianum* variant, *Psidium quadrangularis*, *Psidium molle*, *Psidium guineense*, Seedling of Gushiken Sweet, Seedling of Klom Amporn, Seedling of Pear, and Seedling of 138-T. The clade-4 contained Hisar Safeda, Allahabad Safeda, Allahabad Safeda variant, Seedling of Hong Kong White, Seedling of Pink Acid, Seedling of Thailand Seedless, Guava Seedling-3, Guava Seedling-4, and Guava Seedling-5. Although L-49 considered outliers, as it didn’t group with any other guava genotypes. In this study, genetic distance was also calculated between the Allahabad Safeda & Allahabad Safeda variant, *Psidium cattleianum* & *Psidium cattleianum* variant, and *Psidium pumilum* & *Psidium pumilum* variant, which were 28, 39, and 42, respectively (**Supplementary Figure 5**). The minimum genetic distance was found between the Allahabad Safeda variant and Hisar Safeda (19), whereas the maximum genetic distance was recorded between the Red Diamond and *Psidium cattleianum* variant (186).

### Population structure analysis

**Figure 5:**
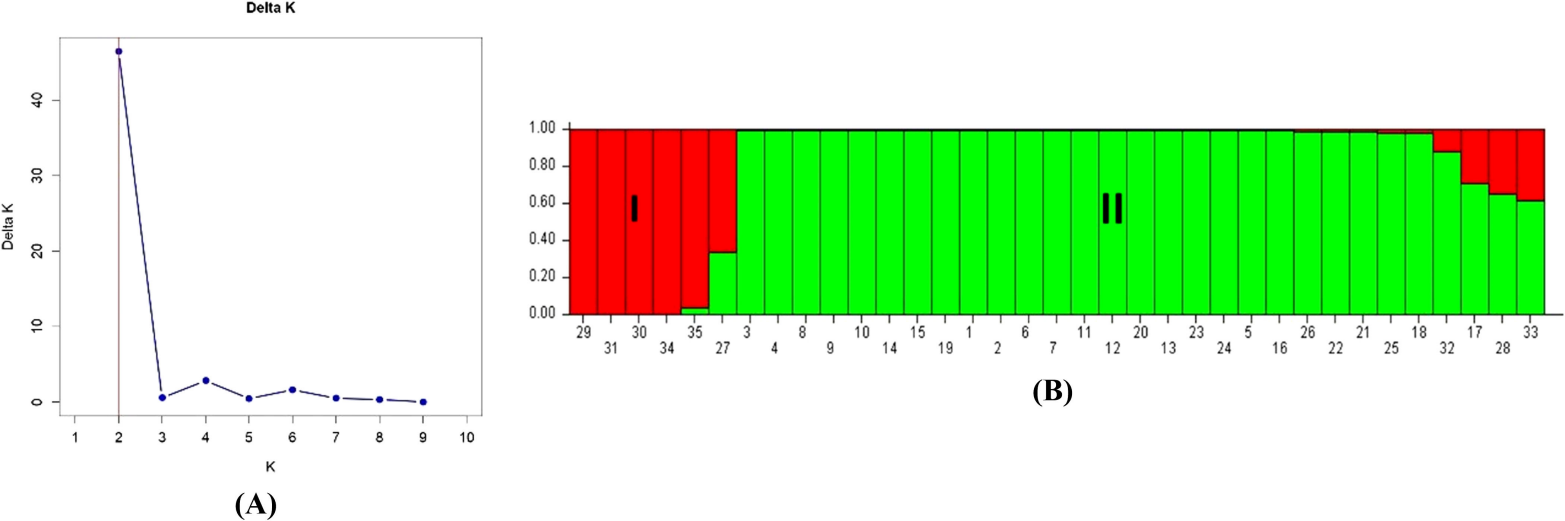
Model based structure analysis. **(A)** Estimation of *Psidium* population using LnP(D) derived Delta K with k ranged from 1 to 10. **(B)** Barplot of population structure (K=2) of 35 selected guava genotypes based on 53 polymorphic g-SSR markers. Here, (1) Allahabad Safeda, (2) Allahabad Safeda Variant, (3) Hisar Safeda, (4) L-49, (5) Shweta, (6) Thai, (7) Pusa Pratiksha, (8) Pant Prabhat, (9) Red Selection, (10) Lalit, (11) Punjab Pink, (12) Hisar Surkha, (13) Red Diamond, (14) Arka Kiran, (15) Pusa Aarushi, (16) Purple Guava, (17) Guava Seedling-1, (18) Guava Seedling-2, (19) Guava Seedling-3, (20) Guava Seedling-4, (21) Guava Seedling-5, (22) Hong Kong White Seedling, (23) Pink Acid Seedling, (24) Thailand Seedless Seedling, (25) Pear Seedling, (26)138-T Seedling, (27) Gushiken Sweet Seedling, (28) Klom Amporn Seedling, (29) *Psidium quadrangularis*, (30) *Psidium cattleianum*, (31) *Psidium cattleianum* variant, (32) *Psidium pumilum*, (33) *Psidium pumilum* variant, (34) *Psidium molle*, (35) *Psidium guineense*.

The structure software v.2.3.4 inferred the population structure of the 35 guava genotypes based on genotypic data of 53 polymorphic g-SSR markers and grouped them into two sub-populations. Here, the Evano method (ΔK value) identified two distinct sub-populations (**Figure 5**). These populations were further classified into pure and admixture types based on membership fractions. When the membership fraction of the genotype is more than 0.8, it is considered a pure type. Otherwise, it is considered an admixture type. The two identified populations, Population I and Population II, encompassed various guava genotypes. Population I consisted of *Psidium cattleianum*, *Psidium cattleianum* Variant, *Psidium guineense*, *Psidium molle*, *Psidium quadrangularis* and Seedling of Gushiken Sweet, with Gushiken Sweet seedling classified as an admixture type. Population II contained the remaining guava genotypes, with Seedling of Klom Amporn, Guava Seedling-1, and *Psidium pumilum* variant classified as admixture types. The population-level allelic frequency divergence between the two populations was 0.1706. The mean values of alpha, Fst_1, and Fst_2 were 0.0695, 0.1400, and 0.4403, respectively.

### AMOVA & PCoA analysis

**Figure 6:**
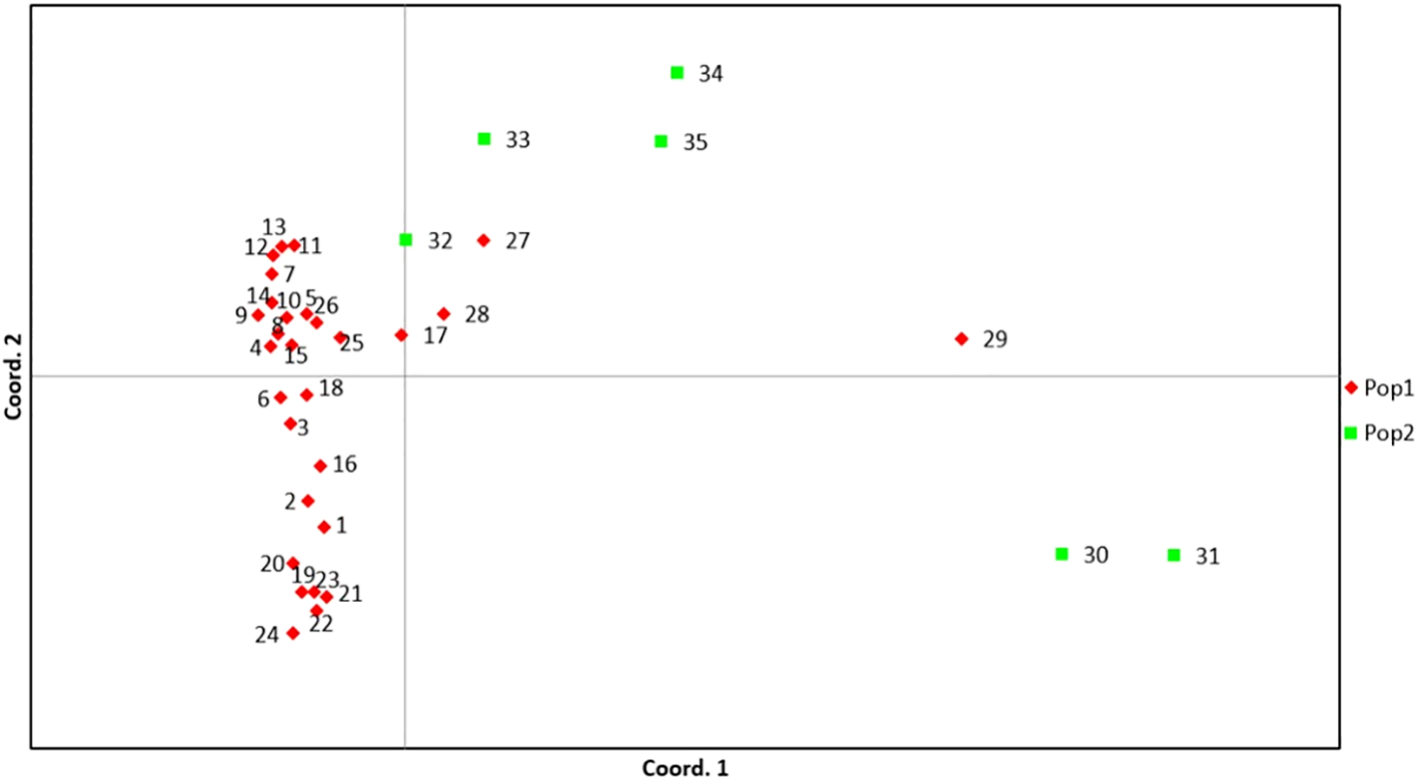
PCoA of 35 selected guava genotypes based on 53 polymorphic g-SSR markers. Here, (1) Allahabad Safeda, (2) Allahabad Safeda Variant, (3) Hisar Safeda, (4) L-49, (5) Shweta, (6) Thai, (7) Pusa Pratiksha, (8) Pant Prabhat, (9) Red Selection, (10) Lalit, (11) Punjab Pink, (12) Hisar Surkha, (13) Red Diamond, (14) Arka Kiran, (15) Pusa Aarushi, (16) Purple Guava, (17) Guava Seedling-1, (18) Guava Seedling-2, (19) Guava Seedling-3, (20) Guava Seedling-4, (21) Guava Seedling-5, (22) Hong Kong White Seedling, (23) Pink Acid Seedling, (24) Thailand Seedless Seedling, (25) Pear Seedling, (26)138-T Seedling, (27) Gushiken Sweet Seedling, (28) Klom Amporn Seedling, (29) *Psidium quadrangularis*, (30) *Psidium cattleianum*, (31) *Psidium cattleianum* variant, (32) *Psidium pumilum*, (33) *Psidium pumilum* variant, (34) *Psidium molle*, (35) *Psidium guineense*.

The analysis of molecular variance (AMOVA) analysis deciphered the molecular variation among populations, among individuals within the population, and within individuals, which were 25%, 52%, and 23%, respectively (P<0.01) (**Table 4**; **Supplementary Figure 6**). The selected guava genotypes demonstrated moderate to high genetic diversity, evident from the first three axes of the principal coordinate analysis (PCoA), which explained 38.63% of the cumulative variance (**Table 5**). The two colours (blue & orange) denote the distinct groupings of guava genotypes in the PCoA that corresponded to the classification of guava genotypes as determined by the model-based population structure approach (**Figure 6**).

**Table 4:** AMOVA for 53 polymorphic g-SSR markers among 35 selected guava genotypes.

Source	df	SS	MS	Est. Var.	%
Among Population	1	87.389	87.389	3.511	25%
Among Individuals	33	579.497	17.561	7.159	52%
Within Individuals	35	113.500	3.243	3.243	23%
Total	69	780.386		13.913	100%

Here, df, Degrees of Freedom; SS, Sum of Squared Deviation; MS, Mean Squared Deviation; Est. Var., Estimated Variance

**Table 5:** Percentage of variation explained by the first 3 axes among 35 selected guava genotypes.

Axis	1	2	3
%	22.40	9.38	6.86
Cum %	22.40	31.77	38.63

### Cross-genera transferability

**Figure 7:**
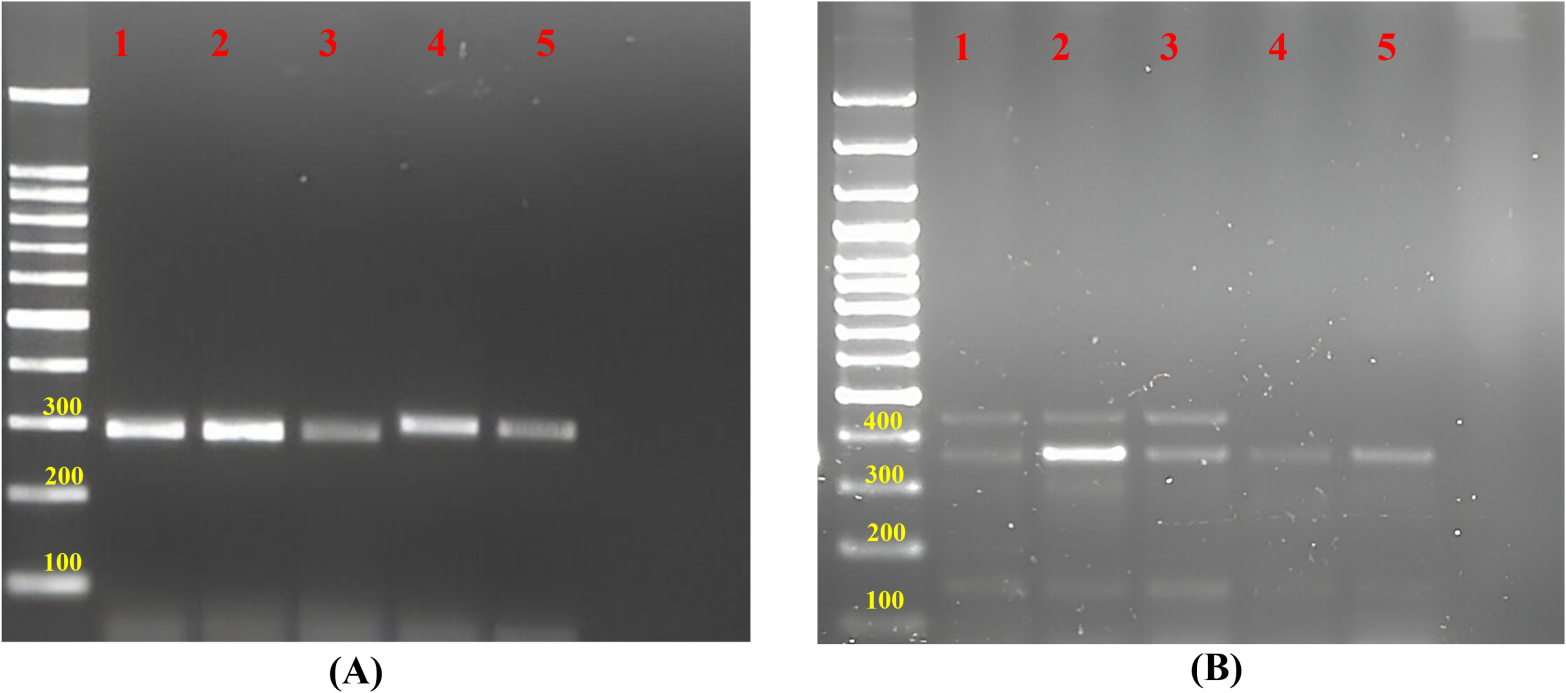
Gel image of amplified products of jamun genotypes for **(A)** FHTGSRR-4.4 and **(B)** FHTGSRR-5.9. Here, (1) *Syzygium cumini* Seedling-1, (2) *Syzygium cumini* Seedling-2, (3) *Syzygium cumini* Seedling-3, (4) *Syzygium fruticosum* Seedling-1, (5) *Syzygium fruticosum* Seedling-2.

In this current study, 75 novel g-SSR markers were also screened for five selected jamun (*Syzygium* sp.) genotypes. Of these, only 17 markers (FHTGSSR-3.1, FHTGSSR-3.5, FHTGSSR-4.4, FHTGSSR-4.6, FHTGSSR-4.7, FHTGSSR-5.4, FHTGSSR-5.5, FHTGSSR-5.7, FHTGSSR-5.8, FHTGSSR-5.9, FHTGSSR-6.2, FHTGSSR-6.6, FHTGSSR-6.7, FHTGSSR-6.9, FHTGSSR-7.4, FHTGSSR-7.8, FHTGSSR-7.9) were amplified among selected jamun genotypes. Although FHTGSSR-4.4, FHTGSSR-5.7, FHTGSSR-5.9, and FHTGSSR-6.7 were found polymorphic among selected jamun genotypes and were able to differentiate between two jamun species (**Figure 7**). The amplicon size varied between 280bp (FHTGSSR-4.4) and 1200bp (FHTGSSR-5.4) for selected jamun genotypes.

## Discussion

In India, the majority of the guava genotypes were named based on their fruit’s shape, colour, and pulp, while many other types were named after the regions they originated from. Though identifying and selecting superior guava varieties through the observation of phenotypic traits linked to commercially valuable characteristics remains a favoured method for crop improvement, the phenotypic variation in guava crops is frequently affected by environmental factors, which are challenging to manage (Srivastava and Narasimhan, 1967; Thaipong et al., 2005). Thus, the importance of molecular markers is not only for the diversity analysis of guava genotypes but also for genetic improvement programs. Among molecular markers, SSR markers are valued for their high efficiency, ease of use, reproducibility, co-dominance, and moderate-to-high resolution of alleles polymorphism in agarose gels (Collard et al., 2005; Collard and Mackill, 2008). In the case of guava, limited genomic resources, such as SSR markers and partial genome information, have hindered progress in molecular analysis, genetic improvement, and breeding programs.

### Development of novel g-SSR markers through *in silico* analysis

Using the genome sequence of the cultivar “New Age” (GCA_016432845.1), we analysed 397.8 Mbp of the guava genome and identified 92,404 SSR loci (**Supplementary Table 1**). Although in previous studies, 1,88,183 (Thakur et al., 2021) and 88,941 (Kumar et al., 2023) SSR loci were recognized using MISA (Beier et al., 2017) and Krait software (Du et al., 2018) respectively. However, Thakur et al., 2021 and Kumar et al., 2023 used the genome sequence of “Allahabad Safeda” (GCA_019787385.1) and “Zhenshu” (GCA_002914565.1) as reference genomes for SSR loci identification respectively. Both reported that monomers were the most abundant motif type, followed by dimers and trimers. However, in the present investigation, during SSR identification, the most abundant motif type was found to be dimers (>75%), followed by trimers (>15%) (**Figure 1**). Earlier, a high frequency of dimer motifs was also reported in date palm (Al-Faifi et al., 2016) and pistachio nut (Ziya et al., 2016) among other fruit crops. Within dimers, the “TC” (~13.5%) motif composition was abundant in every chromosome except chromosome-1 and chromosome-11, where the most common motif compositions were “GA” (~13.7%) and “AG” (~12.9%), respectively. Moreover, among trimers, the “AAT” (~1.4%) motif composition was found to be the highest in every chromosome (**Supplementary Figure 2**). However, in 2023, Kumar et al. reported that the most abundant dimer motif and trimer motif were AG and AAG, respectively. In the current study, the highest and lowest number of SSR loci were found on chromosome-3 (10,475) and chromosome-9 (6,696), respectively (**Supplementary Table 1**). Similarly, in the previous study, the highest and lowest number of SSR loci were found on chromosome 3 (10,536) and chromosome 8 (6,293), respectively (Kumar et al., 2023). In this investigation, a total of 87,372 SSR loci were used for SSR primer designing, leading to a total of 75,084 novel genomic SSR markers being generated at a genome-wide level. Conversely, Thakur et al. (2021) obtained 152,367 SSR primer pairs from 188,183 SSR loci using Primer3_core, while Kumar et al. (2023) identified flanking primer pairs for 86,426 out of 88,941 SSR loci using the same software. After *in silico* analyses, a total of 75 novel g-SSR markers at a genome-wide level were selected for genetic diversity, cross-species and cross-genera transferability studies.

### PCR amplifications and visibility of amplified products on simple agarose gel

In the present investigation, out of 75 novel g-SSR (FHTGSSR) markers, a total of 72 markers showed reliable amplifications in gradient PCR. The genotyping process for the novel g-SSR markers developed in this study utilized standard PCR protocols and resolved amplicons on a simple 2.5% agarose gel. Unlike previous methods that used a touchdown PCR program followed by capillary electrophoresis and gene scan analysis for allele sizing (Kanupriya et al., 2011; Kidaha et al., 2015; Kherwar et al., 2018), these new markers streamline the process by significantly reducing PCR runtime and improving allele size precision, with allelic variations easily visible on agarose gel (Singh et al., 2010). However, in this study, three g-SSR markers showed no satisfactory amplification, even after repetitive attempts to optimize the PCR reactions and conditions.

### Molecular diversity studies

In the present study, out of 72 g-SSR markers, 53 markers were polymorphic while 19 markers were monomorphic. The monomorphic patterns observed in this investigation suggest that guava accessions shared similar alleles, supporting earlier findings of genetic diversity studies using microsatellite markers (Risterucci et al., 2005; Kanupriya et al., 2011; Kumar et al., 2020, Kumar et al., 2023). These shared allelic configurations were likely inherited from common ancestors and have remained stable without slippages during recombination, a major factor in the evolution of SSR regions (Selvam et al., 2015). In this current study, the amplified allele size was between 110-620bp for 72 g-SSR markers (**Table 2**). Some markers produced amplicon sizes smaller than the expected size while some SSR markers produced larger amplification sizes than expected allele sizes. Smaller and larger amplicons from g-SSR markers indicate deletion of the genomic region and insertion of the intronic region, respectively (Selvam et al., 2015). This study was also found 3 g-SSR markers (FHTGSSR-2.6, FHTGSSR-6.9, FHTGSSR-11.1) to be multi-allelic. This dispersion of SSRs may be due to slippage events during recombination and multiple crossovers at specific loci (Kumar et al., 2023). Genetic diversity indices are crucial for evaluating the effectiveness of the newly developed g-SSR markers in guava. In this study, 161 alleles were amplified altogether for 53 polymorphic FHTGSSR markers. On average, each primer pair yielded 3.04 alleles, with the number of alleles per primer pair ranging from 2 to 7 among the studied guava genotypes (**Table 3**). Previously, Sitther et al. (2014) reported 178 alleles from 20 mPgCIR markers in 35 guava genotypes; Kumar et al. (2020) amplified 90 alleles using 26 newly developed g-SSR markers in 40 genotypes; and Kumar et al. (2023) found 46 alleles from 21 novel g-SSR markers in 19 genotypes. Moreover, in the present investigation, 22 unique alleles were found using 53 polymorphic g-SSR markers, which indicates that the selected 35 genotypes maintained considerable genetic diversity among them (Potts et al., 2012). In this study, the PIC values of the novel g-SSR markers ranged from 0.057 (FHTGSSR-1.2 & FHTGSSR-10.4) to 0.774 (FHTGSSR-4.5) among the selected 35 guava genotypes. Furthermore, SSR markers are considered highly informative or possess strong discriminatory ability between genotypes when their PIC value is greater than 0.5 (Botstein et al., 1980). Among developed g-SSR markers, 12 (FHTGSSR-2.4, FHTGSSR-3.6, FHTGSSR-3.8, FHTGSSR-4.4, FHTGSSR-4.5, FHTGSSR-4.7, FHTGSSR-5.7, FHTGSSR-6.3, FHTGSSR-6.8, FHTGSSR-6.9, FHTGSSR-7.5 and FHTGSSR-7.6) had PIC values more than 0.5 (**Table 3**). Hence, these novel SSR markers have high discrimination power. Previously, high average PIC *viz.* 0.56 (Kherwar et al., 2018) and 0.46 (Kumar et al., 2020) had reported for 24 and 26 SSR markers among Indian guava genotypes. But, in our current study, we also got 15 g-SSR markers with PIC values between 0.06-0.2, which might be the reason the average PIC value for 53 polymorphic g-SSR markers was 0.331, lower than in previous studies. The guava genotypes analysed in this study showed moderate levels of expected heterozygosity (0.375), while observed heterozygosity was comparatively low (0.128), which indicating a moderate discrepancy between expected and observed values. Sitther et al. (2014) suggested that substantial differences between these two values in guava accessions may point to a strong inbreeding depression effect occurring throughout the crop’s domestication process. Model-based population structure analysis is valuable for conserving and optimizing collected genotypes (Uddin and Boerner, 2008). Recently, it has been used to differentiate the genetic structure of guava genotypes. For example, Kherwar et al. (2018) categorized 36 guava varieties, including wild species, into five genetic groups in India. Similarly, Sitther et al. (2014) utilized this structural model to differentiate guava germplasm at Hawaii’s USDA National Plants Germplasm System. The current study applied model-based population structure analysis to categorize 35 selected guava genotypes, including wild *Psidium* species, into two distinct genetic groups or sub-populations, *viz.* Population I and Population II (**Figure 5**). Generally, two populations are called highly divergent when allele frequency divergence between two populations is more than 0.05 (Kumar et al., 2020). In this investigation, high allele-frequency divergence (0.217) was observed between two population groups, which indicates significant genetic diversity among them. Besides, an alpha value approaching zero signifies that individuals belonging to distinct populations are mostly pure type (Li et al., 2014), whereas an alpha value greater than one indicates that all individuals in a population are admixtures (Ostrowski et al., 2006). In this study, the small alpha value (0.089) suggests a very low number of admixture genotypes in two populations. Besides, AMOVA further demonstrated considerable genetic diversity across populations, among individuals within populations, and within individual genotypes. Earlier, Kherwar et al. (2018) reported a low level of variation (6%) among populations, whereas Kumar et al. (2020) have seen moderate and significant level of genetic variation among population (19%) and among individuals (50%) respectively. Although, in this study, genetic variation was found highest among individuals (55%), whereas moderate level (23%) variation was found among populations (**Supplementary Figure 6**). Additionally, the PCoA also confirmed the genetic clustering of the guava genotypes, including the wild species. In population II, all genotypes were scattered, indicating their genetic distinctiveness from others (**Figure 6**).

### Phylogenetic relationship studies

Phylogenetic studies were conducted in this investigation by constructing an N-J tree that placed the selected 35 guava accessions into two major clusters. The major clusters were further simplified into four distinct clades and one outgroup, indicating a substantial level of genetic diversity among the selected guava genotypes (**Figure 4**). It was elucidated that the studied wild *Psidium* species were grouped into clade-3. Additionally, Seedling of Gushiken Sweet, Klom Amporn, Pear, and 138-T also fell under clade 3, indicating that these exotic varieties are closely phylogenetically related to wild *Psidium* species. The constructed N-J tree also indicated that the Allahabad Safeda variant is closely related to the cultivar Allahabad Safeda, similar to the *Psidium cattleianum* variant being closely connected with *Psidium cattleianum*, and the *Psidium pumilum* variant with *Psidium pumilum*, respectively. Therefore, the newly developed g-SSR loci effectively distinguished the distinctiveness of genetically diverse guava genotypes and species. However, these genomic SSR loci did not categorize the guava genotypes based on their pulp colour. Earlier, Kanupriya et al. (2011); Kherwar et al. (2018), and Kumar et al. (2023) also grouped Indian guava genotypes into two phylogenetic clusters. In 2020, Kumar et al. grouped Indian guava genotypes into two phylogenetic clusters and six clades, which is similar to the findings of the present study.

### Cross-species transferability studies

Among 72 novel validated genome-wide SSR markers, 28 markers were amplified to all the selected *Psidium* wild species. Although in this current study, cross-species transferability was varied from 45.83% (*P. Cattleianum* variant) to 90.28% (*P pumilum* variant). Whereas Kumar et al. (2020) and Kumar et al. (2023) reported that cross-species transferability for their developed novel SSR markers were 38.46-80.77% and 35%, respectively, among selected wild *Psidium* species. In 1998, Peakall et al. noted that the cross-transferability of SSRs among species within the same genus can range from 50% to 100%. In this investigation, the moderate to high cross-species transferability rate for the newly developed g-SSRs indicated that studied wild *Psidium* species are evolutionarily related to the cultivated guava genotypes (*P. guajava* L.). These novel g-SSR markers efficiently identified species-specific and cultivar-specific alleles, serving as unique molecular signatures for each species or cultivar. Although cp-DNA markers were generally found effective for species-level discrimination (Yan et al., 2018), the newly developed g-SSR markers could also differentiate between wild *Psidium* species successfully. Thus, these markers could be used to verify interspecific hybrids and aid in selecting scions and rootstocks for breeding programs.

### Cross-genera transferability studies

In 2013, Rai et al. (2013) investigated the applicability of 23 SSR primer pairs, which were originally developed for *Psidium guajava*, to *Eucalyptus citriodora*, *Eucalyptus camaldulensis*, *Callistemon lanceolatus*, and *Syzygium aromaticum*, all belonging to the Myrtaceae family. Out of the examined loci, over 78.2% of the 23 SSR loci were shown to amplify across different genera in Eucalyptus citriodora, 60.8% in *Eucalyptus camaldulensis*, and 73.9% in both *Callistemon lanceolatus* and *Syzygium aromaticum*. All four chosen species showed transferability for eight markers. However, the cross-genera transferability of SSR markers developed from the guava genome to jamun has not been reported earlier. This investigation also studied cross-genera transferability among selected two jamun species viz., *S*. *cumini*, *S*. *fruticosum*, using FHTGSSR markers. A total of 17 novel g-SSR markers were amplified among both jamun species, of which only four markers (FHTGSSR-4.4, FHTGSSR-5.7, FHTGSSR-5.9, and FHTGSSR-6.7) were able to differentiate between the two jamun species, explicitly demonstrating the cross-genera transferability.

## Conclusion

GMATA software offers comprehensive benefits for rapid SSR mining, SSR analysis, graphical result visualization, primer pair development by flanking identified SSRs, and polymorphism screening using the e-PCR algorithm. Using GMATA, 397.8 Mbp of the guava genome sequence was analysed, and 92,404 SSR loci were identified. The present set of 53 polymorphic g-SSR markers has proven informative and valuable for guava diversity studies. Among these markers, 12 markers demonstrated high discrimination power with PIC values exceeding 0.5, while 19 additional markers showed moderate informativeness with PIC values ranging from 0.3 to 0.5. These markers are not only useful for hybridity confirmation and marker-assisted selection but also exhibit cross-species transferability within *Psidium* spp. and cross-genera transferability among *Syzygium* spp., making them valuable resources for genetics and breeding studies for both guava and jamun.

## Data Availability

The datasets presented in this study can be found in online repositories. The names of the repository/repositories and accession number(s) can be found below: https://www.ncbi.nlm.nih.gov/, GCA_016432845.1.
