# Supplementary material for: Development of genome-wide SSR markers through in silico mining of guava (Psidium guajava L.) genome for genetic diversity analysis and transferability studies across species and genera
Source: Front Plant Sci. 2025 Apr 25;16:1527866. doi: 10.3389/fpls.2025.1527866 (PMC12062180; doi:10.3389/fpls.2025.1527866)
Supplement: Supplementary file 1 [file DataSheet1.pdf]

Supplementary File

SUPPLEMENTARY FIGURES:

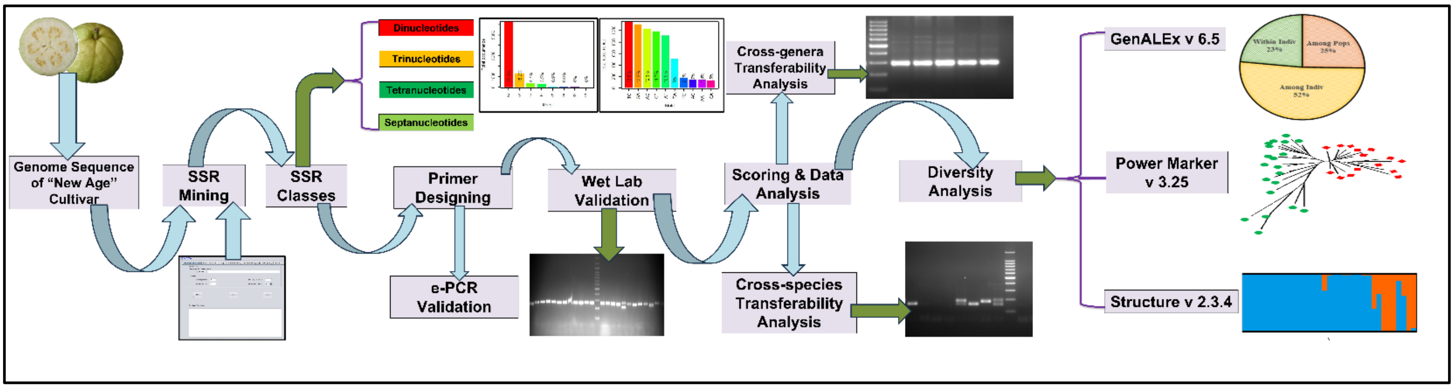

Figure S1. Graphical Abstract

A

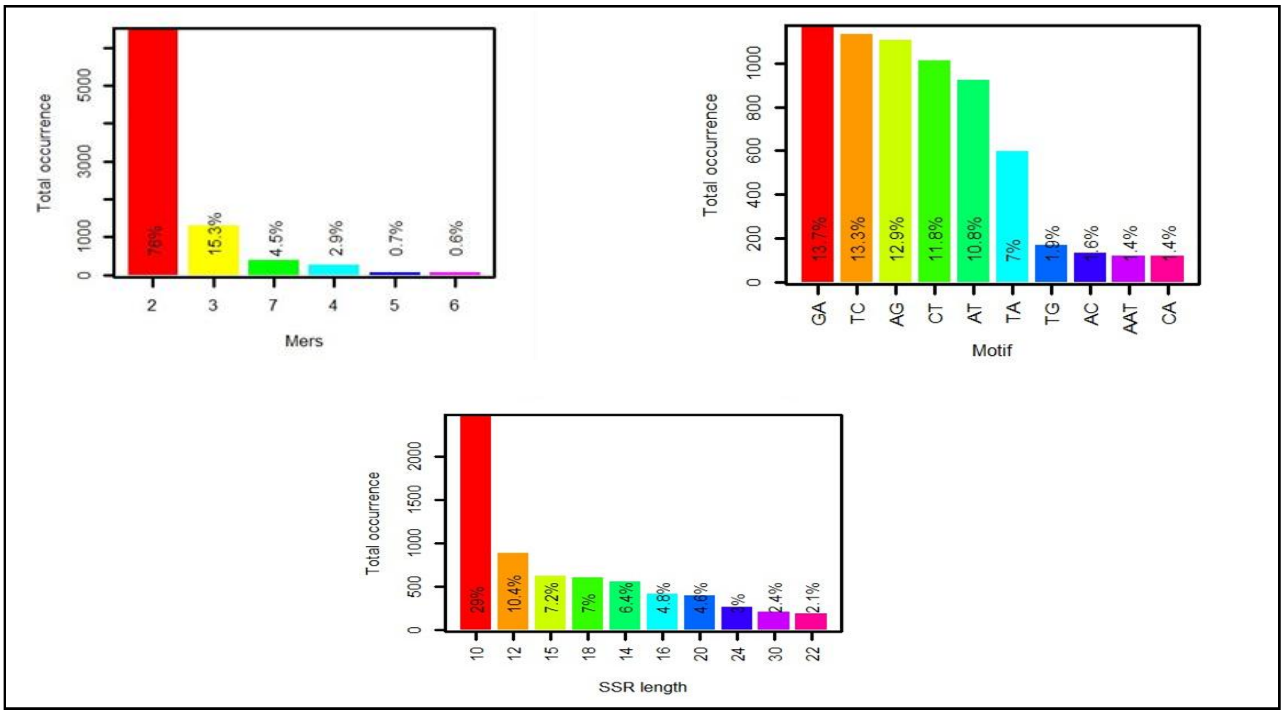
Total occurrence
Mers
2 3 7 4 5 6
76% 15.3% 4.5% 2.9% 0.7% 0.6%
Total occurrence
Motif
GA TC AG CT AT TA TG AC AAT CA
13.7% 13.3% 12.9% 11.8% 10.8% 7% 1.9% 1.6% 1.4% 1.4%
Total occurrence
SSR length
10 12 15 18 14 16 20 24 30 22
29% 10.4% 7.2% 7% 6.4% 4.8% 4.6% 3% 2.4% 2.1%

B

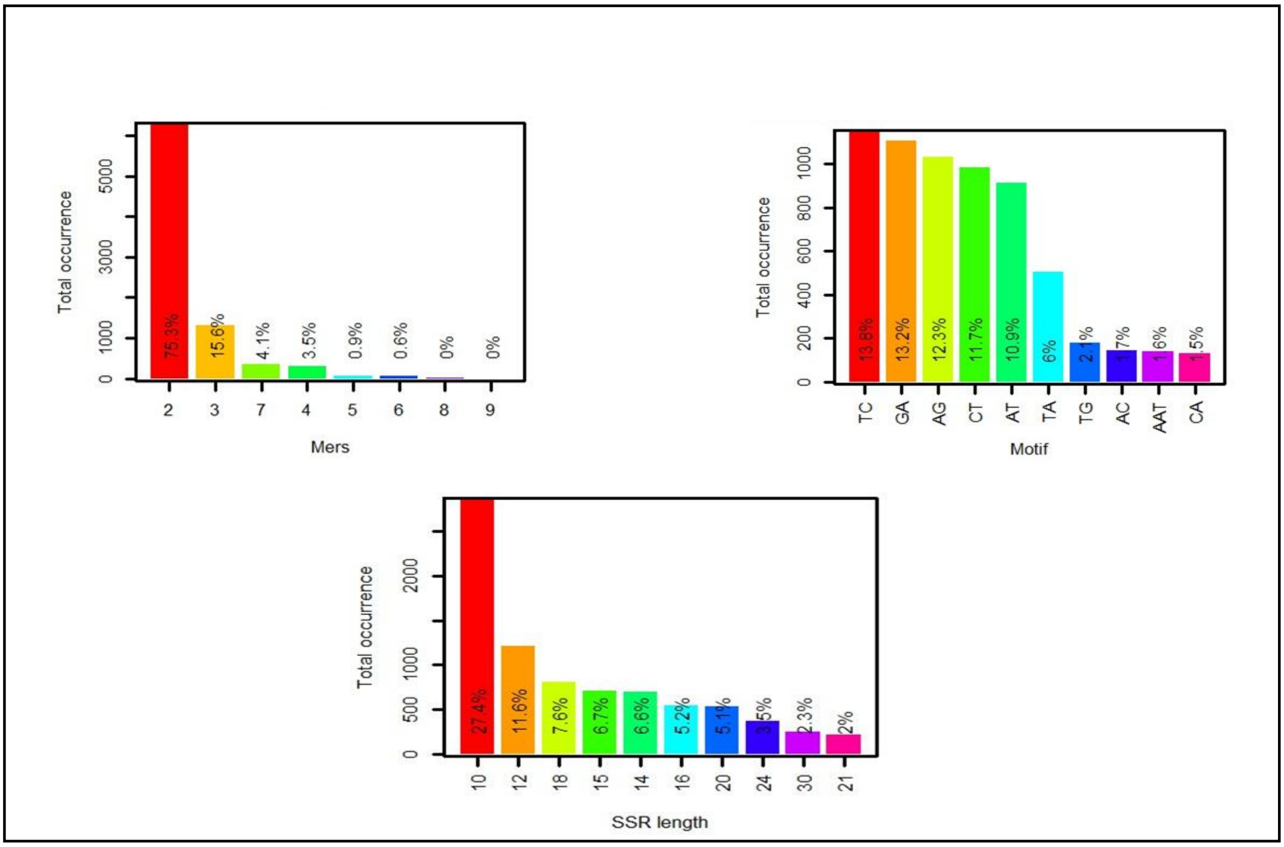
Total occurrence
Mers
2 3 7 4 5 6 8 9
75.3% 15.6% 4.1% 3.5% 0.9% 0.6% 0% 0%
Total occurrence
Motif
TC GA AG CT AT TA TG AC AAT CA
13.8% 13.2% 12.3% 11.7% 10.9% 6% 2.1% 1.7% 1.6% 1.5%
Total occurrence
SSR length
10 12 18 15 14 16 20 24 30 21
27.4% 11.6% 7.6% 6.7% 6.6% 5.2% 5.1% 3.5% 2.3% 2%

C

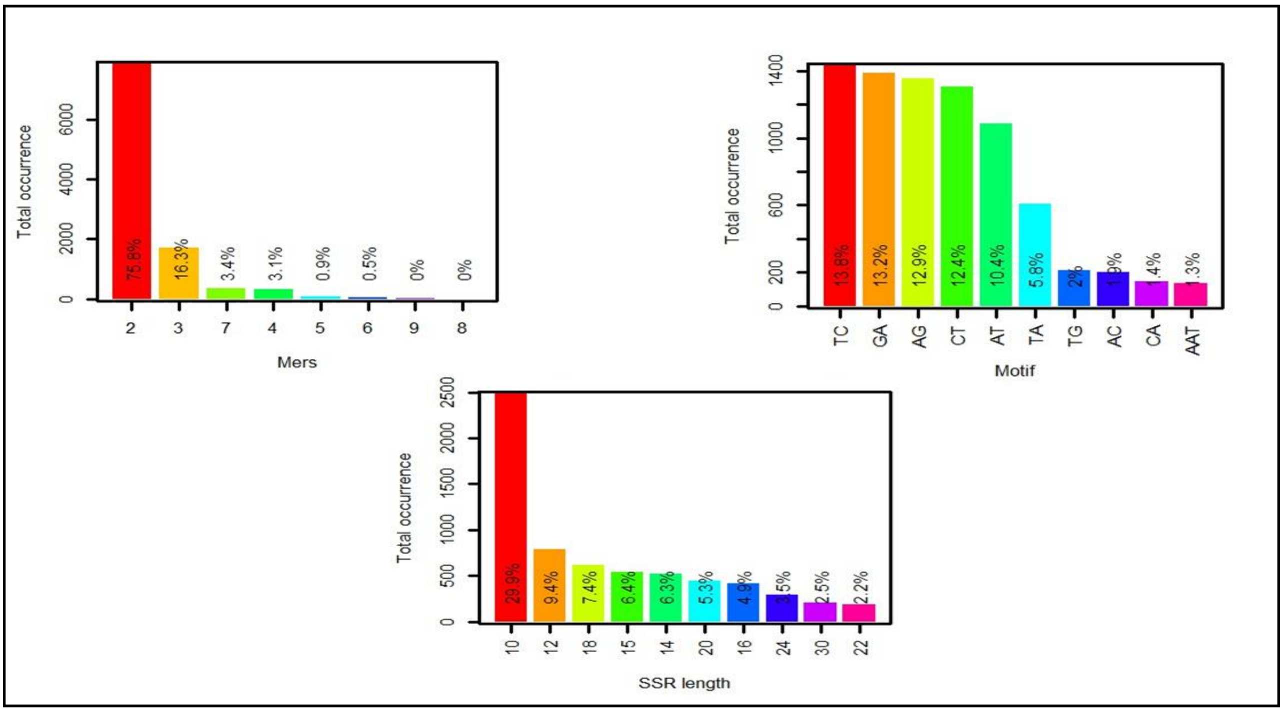

Total occurrence
0
2000
4000
6000
75.8%
16.3%
3.4%
3.1%
0.9%
0.5%
0%
0%
2 3 7 4 5 6 9 8
Mers
Total occurrence
0
200
600
1000
1400
13.8%
13.2%
12.9%
12.4%
10.4%
5.8%
TC GA AG CT AT TA TG AC CA AAT
Motif
Total occurrence
0
500
1000
1500
2000
2500
29.9%
9.4%
7.4%
6.4%
6.3%
5.3%
2.5%
2.2%
10 12 18 15 14 20 16 24 30 22
SSR length

D

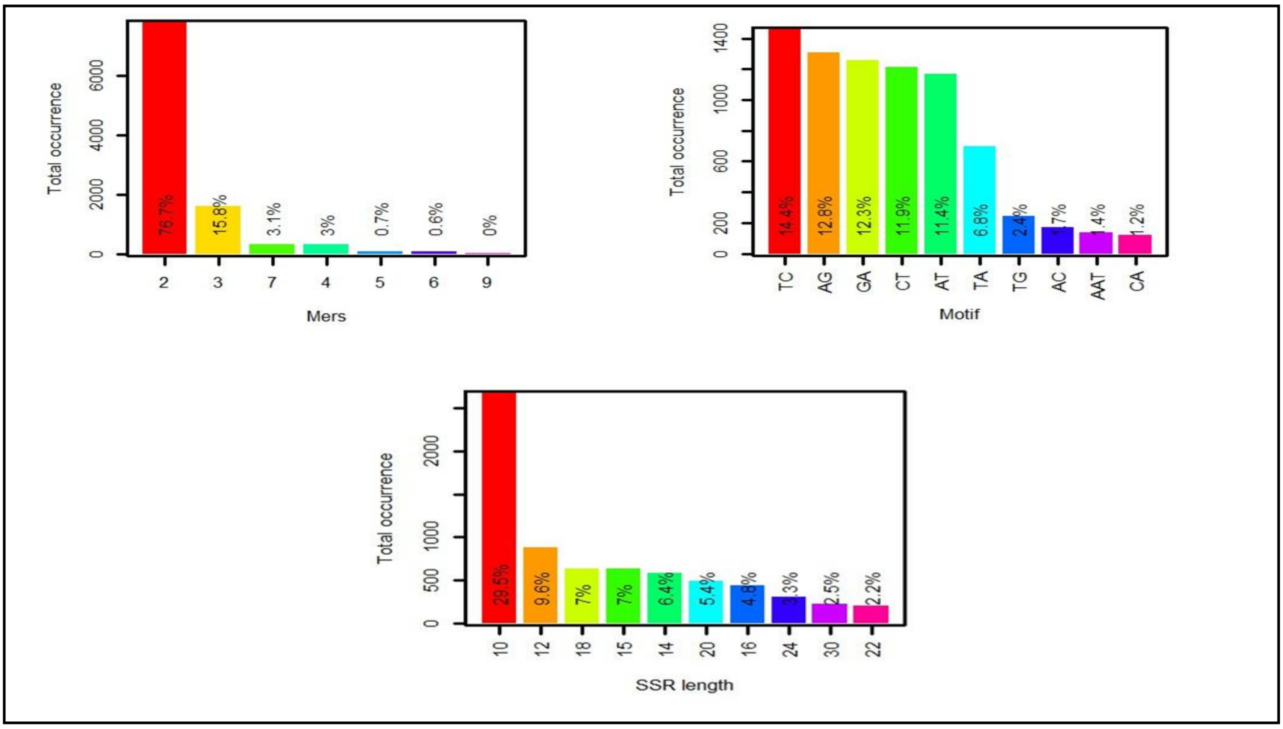

Total occurrence
0
2000
4000
6000
76.7%
15.8%
3.1%
3%
0.7%
0.6%
0%
2 3 7 4 5 6 9
Mers
Total occurrence
0
200
600
1000
1400
14.4%
12.8%
12.3%
11.9%
11.4%
6.8%
TC AG GA CT AT TA TG AC AAT CA
Motif
Total occurrence
0
500
1000
2000
29.5%
9.6%
7%
7%
6.4%
5.4%
2.5%
2.2%
10 12 18 15 14 20 16 24 30 22
SSR length

E

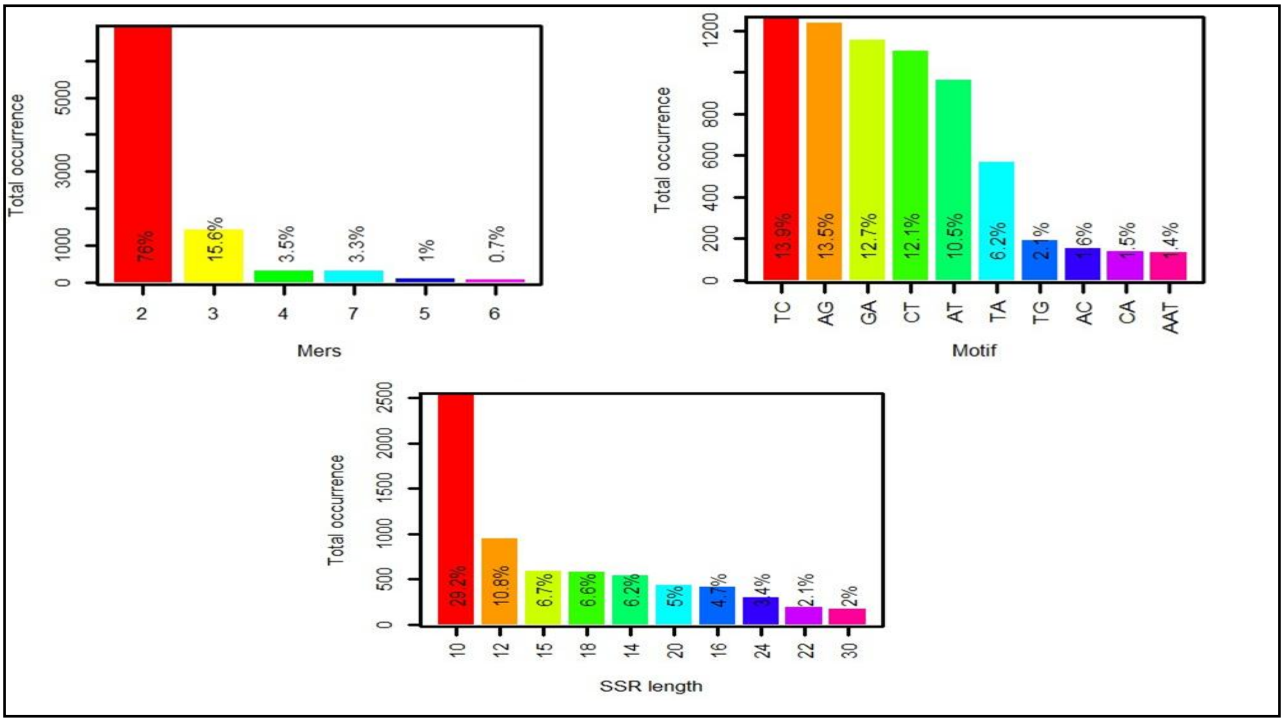
Total occurrence
0
1000
3000
5000
76%
15.6%
3.5%
3.3%
1%
0.7%
2 3 4 7 5 6
Mers
Total occurrence
0
200
400
600
800
1200
13.9%
13.5%
12.7%
12.1%
10.5%
6.2%
TC AG GA CT AT TA TG AC CA AAT
Motif
Total occurrence
0
500
1000
1500
2000
2500
29.2%
10.8%
6.7%
6.6%
6.2%
5%
4.7%
2.1%
2%
10 12 15 18 14 20 16 24 22 30
SSR length

F

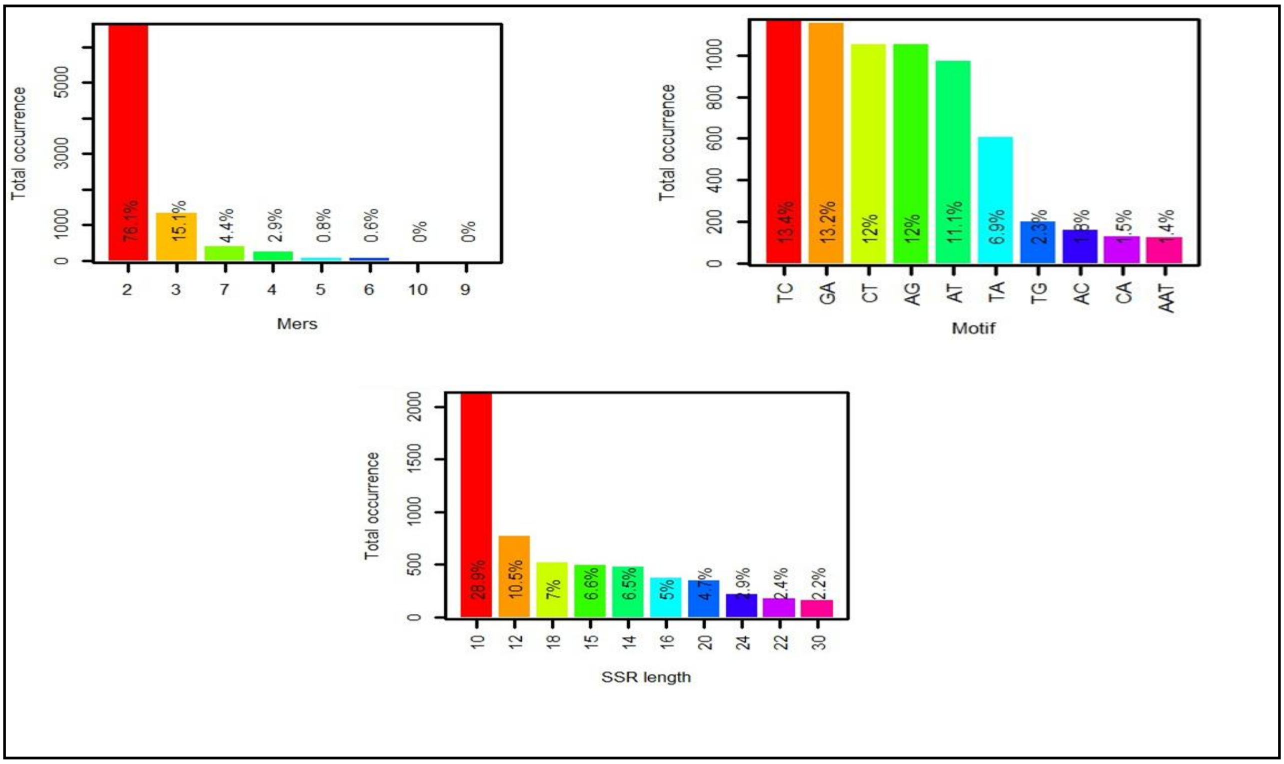
Total occurrence
0
1000
3000
5000
76.1%
15.1%
4.4%
2.9%
0.8%
0.6%
0%
0%
2 3 7 4 5 6 10 9
Mers
Total occurrence
0
200
400
600
800
1000
13.4%
13.2%
12%
12%
11.1%
6.9%
1.4%
TC GA CT AG AT TA TG AC CA AAT
Motif
Total occurrence
0
500
1000
1500
2000
28.9%
10.5%
7%
6.6%
6.5%
5%
4.7%
2.4%
2.2%
10 12 18 15 14 16 20 24 22 30
SSR length

G

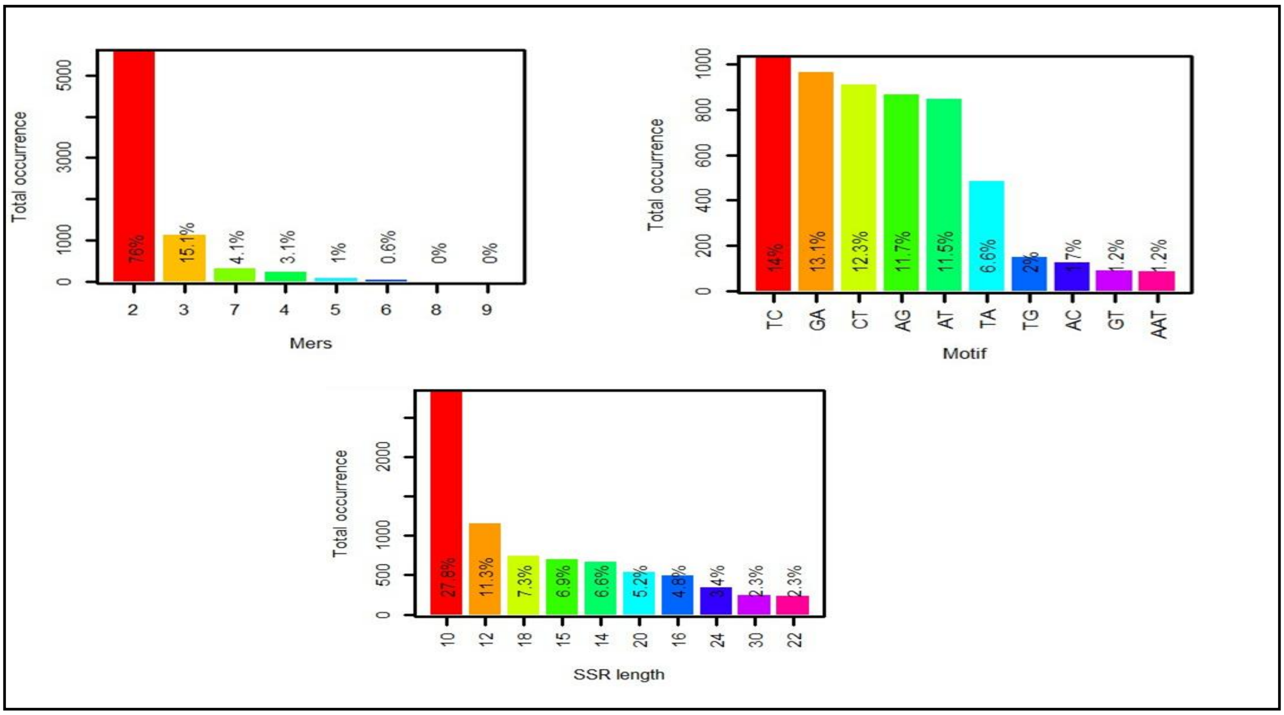
Total occurrence
0
1000
3000
5000
76%
15.1%
4.1%
3.1%
1%
0.6%
0%
0%
2 3 7 4 5 6 8 9
Mers
Total occurrence
0
200
400
600
800
1000
14%
13.1%
12.3%
11.7%
11.5%
6.6%
2%
1.7%
1.2%
1.2%
TC GA CT AG AT TA TG AC GT AAT
Motif
Total occurrence
0
500
1000
2000
27.8%
11.3%
7.3%
6.9%
6.6%
5.2%
4.8%
3.4%
2.3%
2.3%
10 12 18 15 14 20 16 24 30 22
SSR length

H

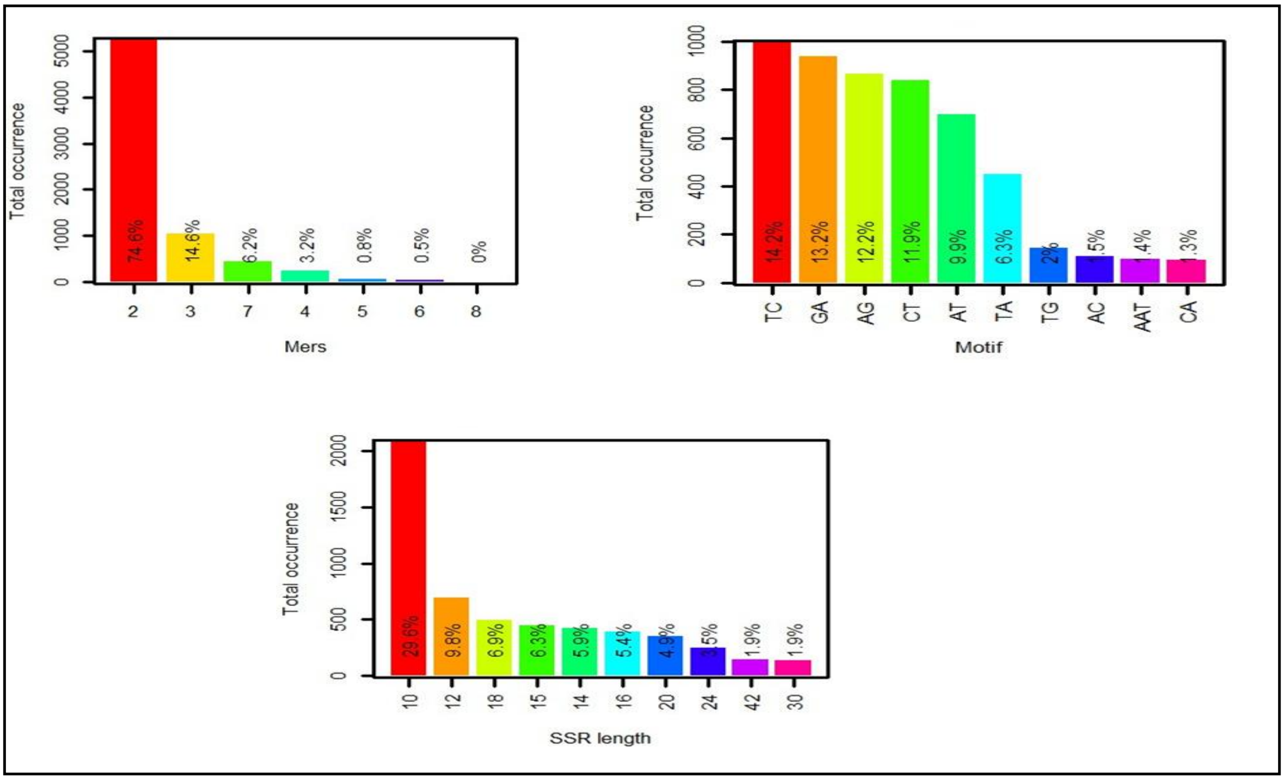
Total occurrence
0
1000
2000
3000
4000
5000
74.6%
14.6%
6.2%
3.2%
0.8%
0.5%
0%
2 3 7 4 5 6 8
Mers
Total occurrence
0
200
400
600
800
1000
14.2%
13.2%
12.2%
11.9%
9.9%
6.3%
2%
1.5%
1.4%
1.3%
TC GA AG CT AT TA TG AC AAT CA
Motif
Total occurrence
0
500
1000
1500
2000
29.6%
9.8%
6.9%
6.3%
5.9%
5.4%
4.9%
3.5%
1.9%
1.9%
10 12 18 15 14 16 20 24 42 30
SSR length

I

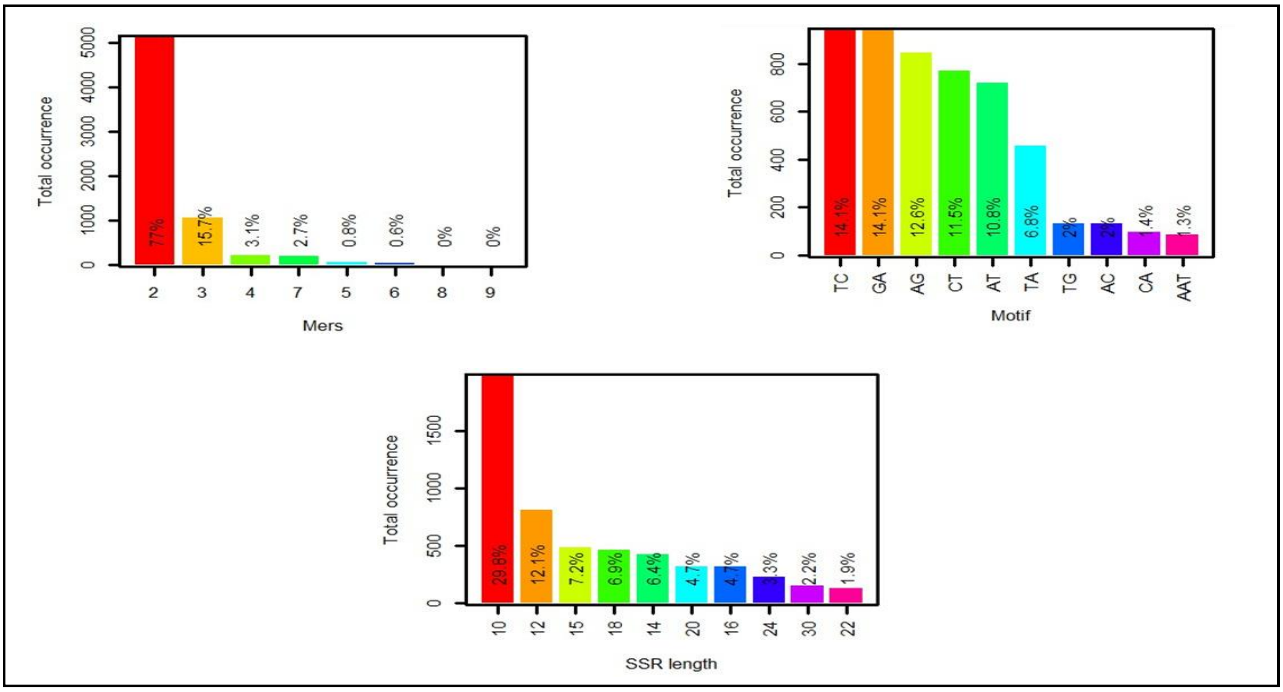

Total occurrence
Mers
2 3 4 7 5 6 8 9
77% 15.7% 3.1% 2.7% 0.8% 0.6% 0% 0%
Total occurrence
Motif
TC GA AG CT AT TA TG AC CA AAT
14.1% 14.1% 12.6% 11.5% 10.8% 6.8% 2% 2% 1.4% 1.3%
Total occurrence
SSR length
10 12 15 18 14 20 16 24 30 22
29.8% 12.1% 7.2% 6.9% 6.4% 4.7% 4.7% 3.3% 2.2% 1.9%

J

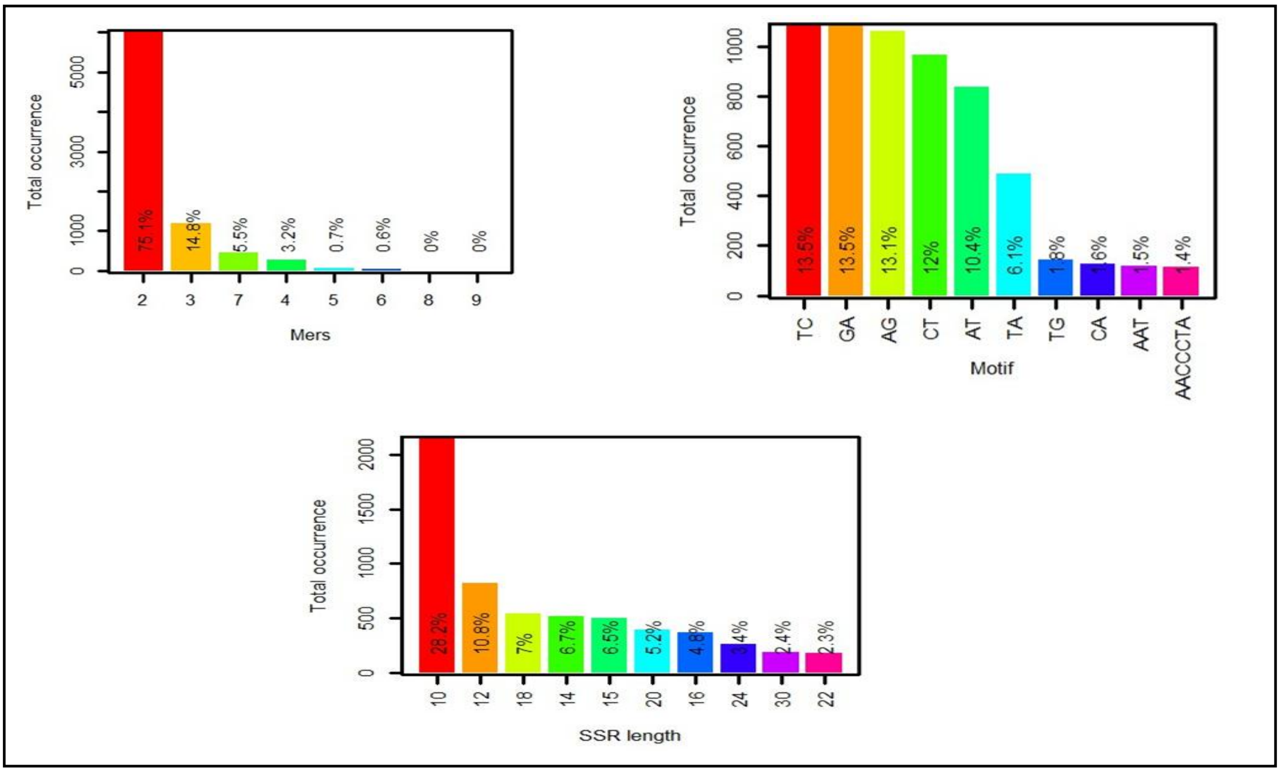

Total occurrence
Mers
2 3 7 4 5 6 8 9
75.1% 14.8% 5.5% 3.2% 0.7% 0.6% 0% 0%
Total occurrence
Motif
TC GA AG CT AT TA TG CA AAT AACCCTA
13.5% 13.5% 13.1% 12% 10.4% 6.1% 1.8% 1.6% 1.5% 1.4%
Total occurrence
SSR length
10 12 18 14 15 20 16 24 30 22
28.2% 10.8% 7% 6.7% 6.5% 5.2% 4.8% 3.4% 2.4% 2.3%

K

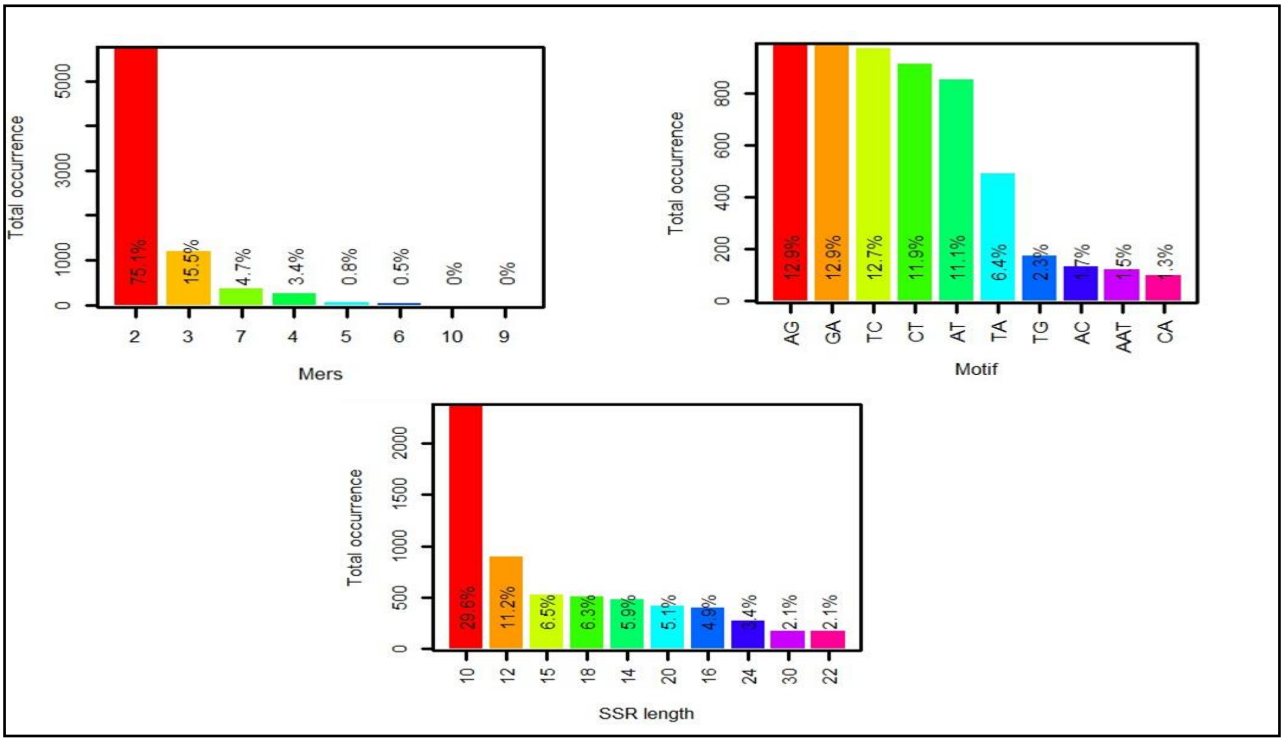

Figure S2. Graphical representation of motif types, motif composition and SSR length for (A) Chromosome-1, (B) Chromosome-2, (C) Chromosome-3, (D) Chromosome-4, (E) Chromosome-5, (F) Chromosome-6, (G) Chromosome-7, (H) Chromosome-8, (I) Chromosome-9, (J) Chromosome-10, (K) Chromosome-11.

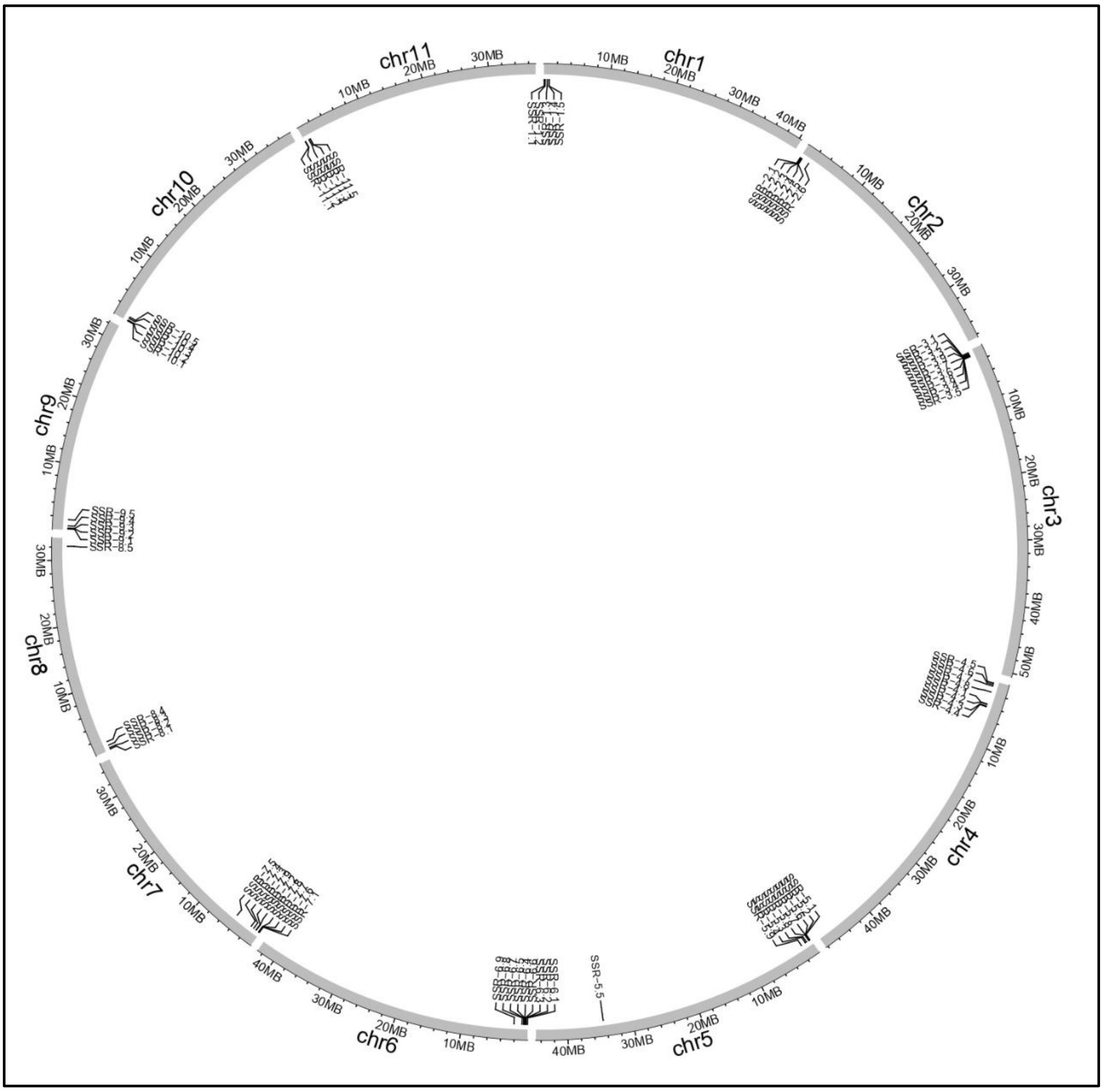

Figure S3. Circos graph indicating chromosome wise distribution of selected 75 g-SSR markers. Here, SSR=FHTGSSR.

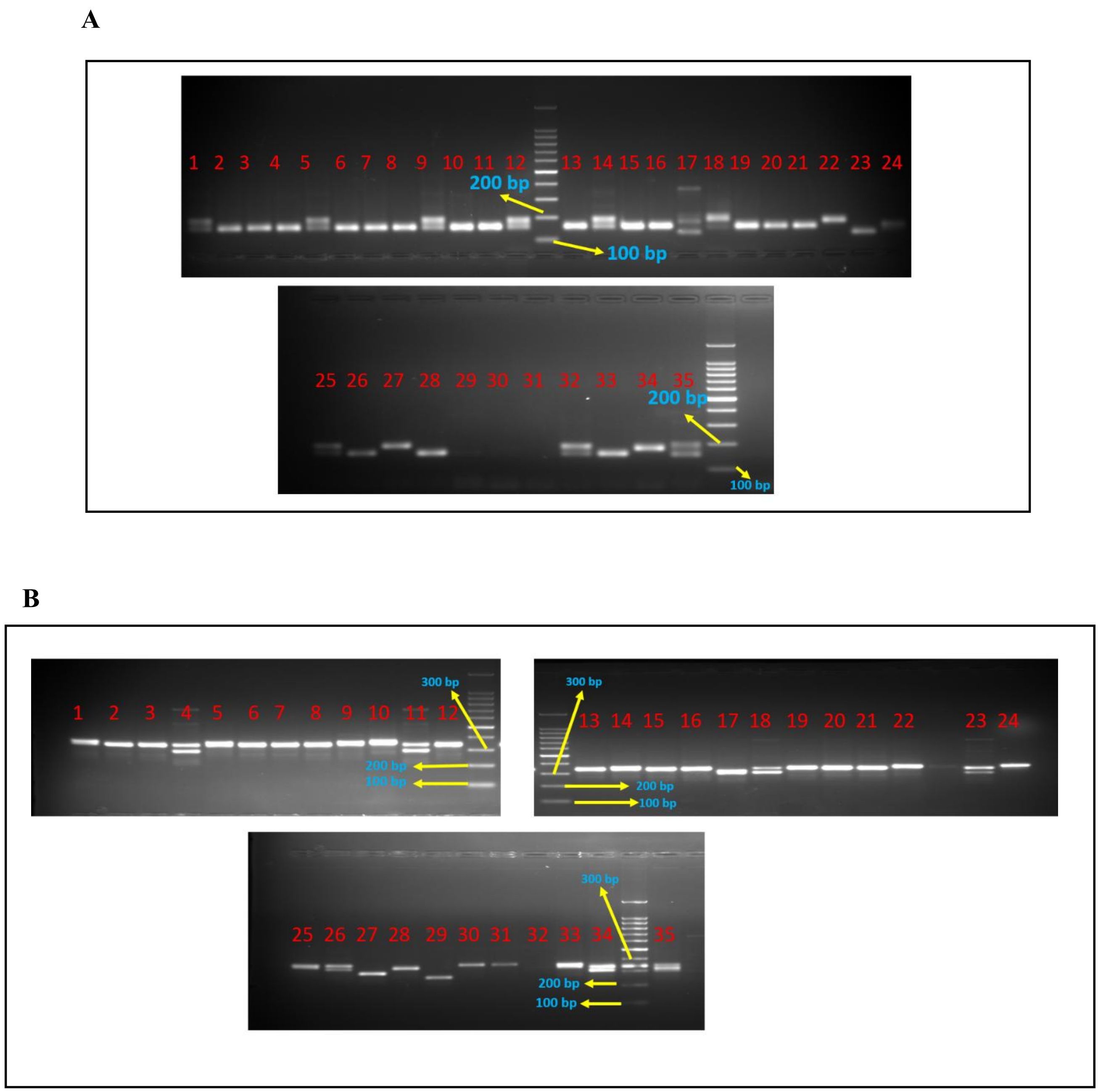

Figure S4. Gel image of amplified products of guava genotypes for (A) FHTGSSR-2.1 and (B) FHTGSSR-2.4.

A	B	C	D	E	F	G	H	I	J	K	L	M	N	O	P	Q	R	S	T	U	V	W	X	Y	Z	AA	AB	AC	AD	AE	AF	AG	AH	AI	
0																																			**A**
25	0																																		**B**
36	**19**	0																																	**C**
47	36	28	0																																**D**
56	50	43	31	0																															**E**
51	46	51	41	36	0																														**F**
55	52	44	27	28	33	0																													**G**
45	48	45	31	44	42	27	0																												**H**
47	38	33	23	32	28	28	32	0																											**I**
51	51	40	36	35	47	27	29	35	0																										**J**
56	56	44	25	37	39	26	29	30	23	0																									**K**
50	55	44	35	36	43	33	27	23	31	22	0																								**L**
66	61	46	36	59	55	39	41	36	44	36	34	0																							**M**
61	48	45	35	44	48	30	48	24	51	40	35	44	0																						**N**
69	63	53	51	47	64	40	58	52	44	48	40	55	34	0																					**O**
62	45	50	48	67	55	56	73	51	74	66	70	72	39	39	0																				**P**
65	68	64	58	58	70	42	54	65	53	58	64	69	53	62	75	0																			**Q**
60	63	64	51	59	67	42	59	61	62	50	56	51	45	43	58	56	0																		**R**
48	34	42	51	62	43	58	61	44	54	64	58	68	60	53	46	85	70	0																	**S**
56	40	35	36	47	54	50	54	45	50	53	60	53	55	58	51	76	52	32	0																**T**
58	59	62	54	67	67	64	59	65	64	64	64	64	73	75	72	83	58	54	37	0															**U**
37	60	55	59	68	60	71	58	56	65	63	51	69	64	70	75	77	69	43	52	55	0														**V**
48	58	59	55	57	60	60	54	56	60	58	58	73	62	70	83	70	61	53	44	45	42	0													**W**
48	52	52	54	62	48	56	64	52	55	60	69	67	62	58	63	75	60	31	36	51	40	36	0												**X**
60	72	60	65	58	62	59	64	54	51	51	37	53	66	50	83	88	75	57	78	75	48	68	54	0											**Y**
46	52	35	43	47	51	51	57	39	43	38	44	48	49	47	54	73	74	56	66	78	59	72	53	31	0										**Z**
80	85	76	73	80	89	70	85	65	83	75	70	88	73	81	92	94	73	88	87	94	94	81	86	79	78	0									**AA**
76	65	64	62	79	63	72	81	55	80	72	74	76	79	87	78	107	96	60	79	98	93	93	77	75	64	84	0								**AB**
151	156	149	141	144	160	149	146	151	146	139	143	151	149	156	165	145	145	160	144	147	148	142	147	142	144	132	145	0							**AC**
134	135	138	141	139	143	144	147	143	142	142	146	156	149	149	144	138	156	140	145	156	147	145	147	150	142	144	140	108	0						**AD**
168	169	172	175	174	175	176	177	177	172	172	176	**186**	181	177	174	164	184	172	177	176	179	173	179	176	172	172	169	121	39	0					**AE**
70	66	61	49	65	62	65	66	57	61	55	62	55	65	82	85	92	83	73	70	83	82	76	82	75	61	99	85	134	149	169	0				**AF**
76	70	67	64	75	78	73	74	67	65	53	57	63	69	75	89	100	89	83	89	97	86	86	96	64	59	83	71	131	137	159	42	0			**AG**
112	112	107	107	93	106	96	110	100	105	95	94	113	102	107	115	89	110	115	111	127	118	119	119	107	103	87	109	127	135	157	106	92	0		**AH**
102	93	94	95	99	103	93	97	92	104	100	99	96	106	108	104	103	107	100	98	120	116	125	120	110	108	101	98	134	134	146	97	87	74	0	**AI**

Figure S5. Genetic distance between 35 selected guava genotypes. Here, (A) Allahabad Safeda, (B) Allahabad Safeda Variant, (C) Hisar Safeda, (D) L-49, (E) Shweta, (F) Thai, (G) Pusa Pratiksha, (H) Pant Prabhat, (I) Red Selection, (J) Lalit, (K) Punjab Pink, (L) Hisar Surkha, (M) Red Diamond, (N) Arka Kiran, (O) Pusa Aarushi, (P) Purple Guava , (Q) Guava Seedling-1, (R) Guava Seedling-2, (S) Guava Seedling-3, (T) Guava Seedling-4, (U) Guava Seedling-5, (V) Hong Kong White Seedling, (W) Pink Acid Seedling, (X) Thailand Seedless Seedling, (Y) Pear Seedling, (Z) 138-T Seedling, (AA)=Gushiken Sweet Seedling, (AB)=Klom Amporn Seedling, (AC)=*Psidium quadrangularis*, (AD)=*Psidium cattleianum*, (AE)=*Psidium cattleianum* variant, (AF)=*Psidium pumilum*, (AG)=*Psidium pumilum* variant, (AH)=*Psidium molle*, (AI)=*Psidium guineense* .

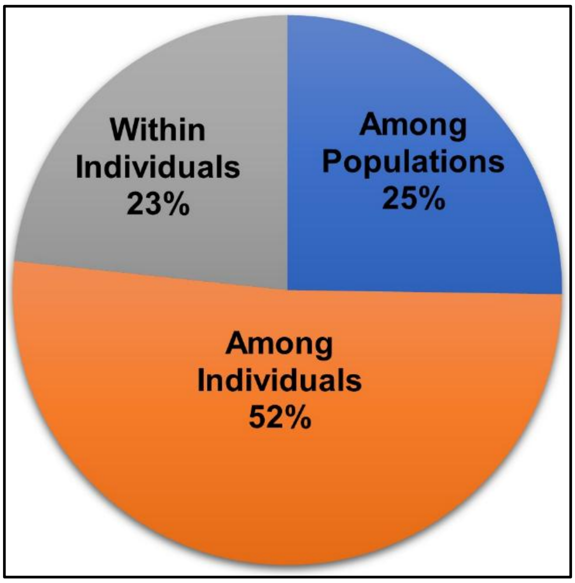

Figure S6. Percentage of molecular variance based on 53 polymorphic g-SSR markers.

SUPPLEMENTARY TABLES:

Table S1. Chromosome wise SSR mining

Chromosome Number	Total Length (bp) Read after formatted	Total Chunking Sites Found after Formatted	Total Number of SSR Loci
**1**	40370300	14	8564
**2**	38465871	13	8377
**3**	50577630	17	10475
**4**	48287879	17	10239
**5**	44769864	15	9136
**6**	42823316	15	8734
**7**	35363649	12	7382
**8**	33379055	12	7080
**9**	32331238	11	6696
**10**	37879573	13	8055
**11**	37021556	13	7666

Table S2. Information of markers developed by GMATA along with e-PCR results

Chromosome Number	Total SSR Loci with Primer Pair Designed	Total SSR Loci without Primer Pair Designed	Total Number of Unique Markers	Total Amplicons e-PCRed from Mapped Markers	Average Amplicons Per Mapped Marker
**1**	8080	484	6986	18454	2.64
**2**	7876	501	6621	19171	2.90
**3**	10029	446	8613	24359	2.83
**4**	9788	451	8505	27216	3.20
**5**	8709	427	7519	21243	2.83
**6**	8248	486	7063	18999	2.69
**7**	6990	392	6021	15853	2.63
**8**	6545	535	5595	14979	2.68
**9**	6431	265	5489	15038	2.74
**10**	7488	567	6375	17379	2.73
**11**	7188	478	6297	17207	2.73

Table S3. List of amplified markers in wild *Psidium* species

Wild *Psidium* Species	Amplified Markers	Total
Psidium quandrancularis	FHTGSSR-1.2, FHTGSSR-1.4, FHTGSSR-2.3, FHTGSSR-2.4, FHTGSSR-2.5, FHTGSSR-2.6, FHTGSSR-3.1, FHTGSSR-3.2, FHTGSSR-3.4, FHTGSSR-3.5, FHTGSSR-3.7, FHTGSSR-3.8, FHTGSSR-3.9, FHTGSSR-4.3, FHTGSSR-4.4, FHTGSSR-4.5, FHTGSSR-4.6, FHTGSSR-4.7, FHTGSSR-4.8, FHTGSSR-5.1, FHTGSSR-5.4, FHTGSSR-5.5, FHTGSSR-5.6, FHTGSSR-5.7, FHTGSSR-5.8, FHTGSSR-5.9, FHTGSSR-6.1, FHTGSSR-6.2, FHTGSSR-6.3, FHTGSSR-6.4, FHTGSSR-6.5, FHTGSSR-6.6, FHTGSSR-6.7, FHTGSSR-6.8, FHTGSSR-6.9, FHTGSSR-7.1, FHTGSSR-7.3, FHTGSSR-7.4, FHTGSSR-7.5, FHTGSSR-7.6, FHTGSSR-7.7, FHTGSSR-7.8, FHTGSSR-7.9, FHTGSSR-8.1, FHTGSSR-8.2, FHTGSSR-8.4, FHTGSSR-9.1, FHTGSSR-9.2, FHTGSSR-9.4, FHTGSSR-9.5, FHTGSSR-10.2, FHTGSSR-10.3, FHTGSSR-11.2, FHTGSSR-11.3, FHTGSSR-11.5.	55
Psidium cattleianum	FHTGSSR-2.2, FHTGSSR-2.3, FHTGSSR-2.4, FHTGSSR-2.6, FHTGSSR-3.1, FHTGSSR-3.2, FHTGSSR-3.4, FHTGSSR-3.5, FHTGSSR-3.7, FHTGSSR-3.8, FHTGSSR-3.9, FHTGSSR-4.1, FHTGSSR-4.4, FHTGSSR-4.5, FHTGSSR-4.6, FHTGSSR-5.1, FHTGSSR-5.4, FHTGSSR-5.5, FHTGSSR-5.6, FHTGSSR-5.7, FHTGSSR-5.8, FHTGSSR-5.9, FHTGSSR-6.1, FHTGSSR-6.2, FHTGSSR-6.6, FHTGSSR-6.7, FHTGSSR-6.9, FHTGSSR-7.1, FHTGSSR-7.3, FHTGSSR-7.4, FHTGSSR-7.5, FHTGSSR-7.7, FHTGSSR-7.9, FHTGSSR-8.2, FHTGSSR-8.4, FHTGSSR-9.1, FHTGSSR-9.2, FHTGSSR-10.2, FHTGSSR-10.3, FHTGSSR-10.4, FHTGSSR-11.2, FHTGSSR-11.3, FHTGSSR-11.5.	43
Psidium cattleianum variant	FHTGSSR-2.2, FHTGSSR-2.3, FHTGSSR-2.4, FHTGSSR-3.1, FHTGSSR-3.2, FHTGSSR-3.4, FHTGSSR-3.5, FHTGSSR-3.8, FHTGSSR-4.1, FHTGSSR-4.4, FHTGSSR-4.5, FHTGSSR-4.6, FHTGSSR-5.1, FHTGSSR-5.4, FHTGSSR-5.5, FHTGSSR-5.6, FHTGSSR-5.7, FHTGSSR-5.8, FHTGSSR-6.2, FHTGSSR-6.6, FHTGSSR-6.7, FHTGSSR-6.9, FHTGSSR-7.1, FHTGSSR-7.4, FHTGSSR-7.5, FHTGSSR-7.9, FHTGSSR-8.2, FHTGSSR-8.4, FHTGSSR-9.1, FHTGSSR-9.2, FHTGSSR-10.3, FHTGSSR-11.3, FHTGSSR-11.5.	33
Psidium pumilum	FHTGSSR-1.2, FHTGSSR-1.4, FHTGSSR-1.5, FHTGSSR-2.1, FHTGSSR-2.2, FHTGSSR-2.3, FHTGSSR-2.4, FHTGSSR-2.5, FHTGSSR-2.6, FHTGSSR-3.1, FHTGSSR-3.2, FHTGSSR-3.3, FHTGSSR-3.4, FHTGSSR-3.5, FHTGSSR-3.8, FHTGSSR-4.1, FHTGSSR-4.2, FHTGSSR-4.3, FHTGSSR-4.4, FHTGSSR-4.5, FHTGSSR-4.6, FHTGSSR-4.7, FHTGSSR-4.8, FHTGSSR-5.1, FHTGSSR-5.4, FHTGSSR-5.5, FHTGSSR-5.6, FHTGSSR-5.7, FHTGSSR-5.8, FHTGSSR-5.9, FHTGSSR-6.1, FHTGSSR-6.2, FHTGSSR-6.3, FHTGSSR-6.4, FHTGSSR-6.5, FHTGSSR-6.6, FHTGSSR-6.7, FHTGSSR-6.8, FHTGSSR-6.9, FHTGSSR-7.1, FHTGSSR-7.2, FHTGSSR-7.3, FHTGSSR-7.4, FHTGSSR-7.5, FHTGSSR-7.6, FHTGSSR-7.7, FHTGSSR-7.8, FHTGSSR-7.9, FHTGSSR-8.1, FHTGSSR-8.4, FHTGSSR-8.5, FHTGSSR-9.1, FHTGSSR-9.2, FHTGSSR-9.3, FHTGSSR-9.4, FHTGSSR-9.5, FHTGSSR-10.2, FHTGSSR-10.3, FHTGSSR-10.4, FHTGSSR-11.1, FHTGSSR-11.2, FHTGSSR-11.3, FHTGSSR-11.4, FHTGSSR-11.5.	64
Psidium pumilum variant	FHTGSSR-1.2, FHTGSSR-1.4, FHTGSSR-1.5, FHTGSSR-2.1, FHTGSSR-2.2, FHTGSSR-2.3, FHTGSSR-2.5, FHTGSSR-2.6, FHTGSSR-3.1, FHTGSSR-3.2, FHTGSSR-3.3, FHTGSSR-3.4, FHTGSSR-3.5, FHTGSSR-3.6, FHTGSSR-3.7, FHTGSSR-3.8, FHTGSSR-4.1, FHTGSSR-4.2, FHTGSSR-4.4, FHTGSSR-4.5, FHTGSSR-4.6, FHTGSSR-4.7, FHTGSSR-4.8, FHTGSSR-5.1, FHTGSSR-5.2, FHTGSSR-5.5, FHTGSSR-5.6, FHTGSSR-5.7, FHTGSSR-5.8, FHTGSSR-5.9, FHTGSSR-6.1, FHTGSSR-6.2, FHTGSSR-6.3, FHTGSSR-6.4, FHTGSSR-6.5, FHTGSSR-6.6, FHTGSSR-6.7, FHTGSSR-6.8, FHTGSSR-6.9, FHTGSSR-7.1, FHTGSSR-7.2, FHTGSSR-7.3, FHTGSSR-7.4, FHTGSSR-7.5, FHTGSSR-7.6, FHTGSSR-7.7, FHTGSSR-7.8, FHTGSSR-7.9, FHTGSSR-8.1, FHTGSSR-8.2, FHTGSSR-8.3, FHTGSSR-8.4, FHTGSSR-8.5, FHTGSSR-9.1, FHTGSSR-9.2, FHTGSSR-9.3, FHTGSSR-9.4, FHTGSSR-9.5, FHTGSSR-10.2, FHTGSSR-10.3, FHTGSSR-10.4, FHTGSSR-11.2, FHTGSSR-11.3, FHTGSSR-11.4, FHTGSSR-11.5.	65
Psidium molle	FHTGSSR-1.2, FHTGSSR-1.4, FHTGSSR-1.5, FHTGSSR-2.1, FHTGSSR-2.2, FHTGSSR-2.3, FHTGSSR-2.4, FHTGSSR-2.5, FHTGSSR-2.6, FHTGSSR-3.1, FHTGSSR-3.2, FHTGSSR-3.3, FHTGSSR-3.4, FHTGSSR-3.5, FHTGSSR-3.6, FHTGSSR-3.7, FHTGSSR-3.8, FHTGSSR-3.9, FHTGSSR-4.1, FHTGSSR-4.2, FHTGSSR-4.4, FHTGSSR-4.5, FHTGSSR-4.6, FHTGSSR-4.8, FHTGSSR-5.1, FHTGSSR-5.2, FHTGSSR-5.4, FHTGSSR-5.5, FHTGSSR-5.6, FHTGSSR-5.8, FHTGSSR-5.9, FHTGSSR-6.1, FHTGSSR-6.2, FHTGSSR-6.3, FHTGSSR-6.4, FHTGSSR-6.6, FHTGSSR-6.7, FHTGSSR-6.8, FHTGSSR-6.9, FHTGSSR-7.1, FHTGSSR-7.3, FHTGSSR-7.4, FHTGSSR-7.5, FHTGSSR-7.6, FHTGSSR-7.7, FHTGSSR-7.8, FHTGSSR-7.9, FHTGSSR-8.1, FHTGSSR-8.2, FHTGSSR-8.4, FHTGSSR-8.5, FHTGSSR-9.1, FHTGSSR-9.2, FHTGSSR-9.4, FHTGSSR-9.5, FHTGSSR-10.2, FHTGSSR-10.3, FHTGSSR-10.4, FHTGSSR-11.1, FHTGSSR-11.2, FHTGSSR-11.3, FHTGSSR-11.5.	62
Psidium guineense	FHTGSSR-1.2, FHTGSSR-1.4, FHTGSSR-1.5, FHTGSSR-2.1, FHTGSSR-2.2, FHTGSSR-2.3, FHTGSSR-2.4, FHTGSSR-2.5, FHTGSSR-3.1, FHTGSSR-3.2, FHTGSSR-3.3, FHTGSSR-3.4, FHTGSSR-3.5, FHTGSSR-3.6, FHTGSSR-3.7, FHTGSSR-3.8, FHTGSSR-4.1, FHTGSSR-4.2, FHTGSSR-4.3, FHTGSSR-4.4, FHTGSSR-4.5, FHTGSSR-4.6, FHTGSSR-4.8, FHTGSSR-5.1, FHTGSSR-5.4, FHTGSSR-5.5, FHTGSSR-5.6, FHTGSSR-5.7, FHTGSSR-5.8, FHTGSSR-5.9, FHTGSSR-6.2, FHTGSSR-6.3, FHTGSSR-6.4, FHTGSSR-6.5, FHTGSSR-6.7, FHTGSSR-6.8, FHTGSSR-6.9, FHTGSSR-7.1, FHTGSSR-7.3, FHTGSSR-7.4, FHTGSSR-7.5, FHTGSSR-7.6, FHTGSSR-7.7, FHTGSSR-7.8,	63

	FHTGSSR-7.9, FHTGSSR-8.1, FHTGSSR-8.2, FHTGSSR-8.3, FHTGSSR-8.4, FHTGSSR-8.5, FHTGSSR-9.1, FHTGSSR-9.2, FHTGSSR-9.3, FHTGSSR-9.4, FHTGSSR-9.5, FHTGSSR-10.2, FHTGSSR-10.3, FHTGSSR-10.4, FHTGSSR-11.1, FHTGSSR-11.2, FHTGSSR-11.3, FHTGSSR-11.4, FHTGSSR-11.5.	
